# The Diapause Lipidomes of Three Closely Related Beetle Species Reveal Mechanisms for Tolerating Energetic and Cold Stress in High-Latitude Seasonal Environments

**DOI:** 10.3389/fphys.2020.576617

**Published:** 2020-09-25

**Authors:** Philipp Lehmann, Melissa Westberg, Patrik Tang, Leena Lindström, Reijo Käkelä

**Affiliations:** ^1^Department of Zoology, Stockholm University, Stockholm, Sweden; ^2^Department of Biological and Environmental Science, University of Jyväskylä, Jyväskylä, Finland; ^3^Molecular and Integrative Biosciences Research Programme, Faculty of Biological and Environmental Sciences, University of Helsinki, Helsinki, Finland; ^4^Department of Biological Sciences, University of Bergen, Bergen, Norway; ^5^Helsinki University Lipidomics Unit, Helsinki Institute for Life Science and Biocenter Finland, Helsinki, Finland

**Keywords:** climate change, range expansion, abiotic stress, invasive species, pest insect

## Abstract

During winter insects face energetic stress driven by lack of food, and thermal stress due to sub-optimal and even lethal temperatures. To survive, most insects living in seasonal environments such as high latitudes, enter diapause, a deep resting stage characterized by a cessation of development, metabolic suppression and increased stress tolerance. The current study explores physiological adaptations related to diapause in three beetle species at high latitudes in Europe. From an ecological perspective, the comparison is interesting since one species (*Leptinotarsa decemlineata*) is an invasive pest that has recently expanded its range into northern Europe, where a retardation in range expansion is seen. By comparing its physiological toolkit to that of two closely related native beetles (*Agelastica alni* and *Chrysolina polita*) with similar overwintering ecology and collected from similar latitude, we can study if harsh winters might be constraining further expansion. Our results suggest all species suppress metabolism during diapause and build large lipid stores before diapause, which then are used sparingly. In all species diapause is associated with temporal shifts in storage and membrane lipid profiles, mostly in accordance with the homeoviscous adaptation hypothesis, stating that low temperatures necessitate acclimation responses that increase fluidity of storage lipids, allowing their enzymatic hydrolysis, and ensure integral protein functions. Overall, the two native species had similar lipidomic profiles when compared to the invasive species, but all species showed specific shifts in their lipid profiles after entering diapause. Taken together, all three species show adaptations that improve energy saving and storage and membrane lipid fluidity during overwintering diapause. While the three species differed in the specific strategies used to increase lipid viscosity, the two native beetle species showed a more canalized lipidomic response, than the recent invader. Since close relatives with similar winter ecology can have different winter ecophysiology, extrapolations among species should be done with care. Still, range expansion of the recent invader into high latitude habitats might indeed be retarded by lack of physiological tools to manage especially thermal stress during winter, but conversely species adapted to long cold winters may face these stressors as a consequence of ongoing climate warming.

## Introduction

Life in high latitude environments with large seasonal variation in abiotic and biotic environmental factors demands optimizing life-history and stress-tolerance according to prevalent conditions (Tauber et al., 1986; Schmidt-Nielsen, 1990). A major challenge is surviving winter, and many adaptations exist to this end, which can be grouped into three main categories: migrating to more benign environments (Dingle, 1978), tolerating the winter in an active state, often through structural, physiological and biochemical acclimatization (Leather, 1993) or spending winter in a resting state (Danks, 1987). Most small ectotherms, such as insects, spend winter in diapause, a pre-programmed deep resting stage, characterized by a cessation of reproductive and ontogenetic development, depressed metabolic rate and increased stress tolerance (Tauber et al., 1986; Denlinger and Lee, 2010; Tougeron, 2019).

One major challenge during diapause is surviving energetic stress. During diapause insects generally do not feed and are thus dependent on stored energy for survival (Hahn and Denlinger, 2007). Stored energy is accumulated prior to diapause, generally in the form of lipids, but to a smaller extent also as carbohydrates and proteins (Hahn and Denlinger, 2011). Energy stores are utilized to fuel basic metabolic needs during overwintering, but also to complete ontogenetic development, for reproductive maturation or for dispersal after overwintering (Koštál, 2006; Hahn and Denlinger, 2007; Sinclair, 2015). Some diapausing insects store significantly more chemical energy than non-overwintering counterparts, suggesting that they budget for winter-related expenses already during development. However, this is not always possible due to e.g. size constraints, which could limit the amount of extra energy that can be stored. Therefore there is a second, not mutually exclusive option, that diapause is associated with extreme metabolic suppression, which limits energy expenditure, often in a temperature-independent fashion (Williams et al., 2012, 2015; Lehmann et al., 2016; Lindestad et al., 2020).

Diapause is also associated with cold stress (Denlinger and Lee, 2010). Low temperatures lead to among other, impaired enzyme function and ion homeostasis, and subzero temperatures are associated with the risk of ice formation, which is lethal without specialized adaptations (Toxopeus and Sinclair, 2018). Another central homeostasis issue is that, without compensating adaptations, decreasing temperatures reduce the viscosity of storage lipid droplets and cellular phospholipid membranes. The consequences would be impaired accessibility of the droplets for enzymatic hydrolysis deliberating fatty acids for energy, and compromised functions of multiple membrane proteins, respectively (Hazel, 1995). To prevent these adverse effects, ectotherms employ various biochemical structural modifications to maintain the optimal semifluid state of lipid membranes and droplets. These include increasing the average degree of lipid unsaturation, decreasing the average length of fatty acids, reshuffling fatty acids to produce new molecular species without affecting individual fatty acid proportions, head group re-structuring or changing the relative amount of cholesterol in membranes (Koštál, 2010). The various ways of biochemical acclimatization of lipid structures to maintain functionality as temperature decreases are often gathered under the umbrella term HVA, which include the HPA and the DPB theory (Sinensky, 1974; Lewis et al., 1989; Hazel, 1995). Central to all is the temperature-dependent dynamism in the lipid bilayer (Zehmer, 2005; Nickels et al., 2019). It should be stressed that there are several mechanisms by which proper lipid composition and physical properties are maintained, and that no clear taxonomic patterns exist, i.e., some animal groups employing only a certain acclimatization response (Koštál, 2010). Instead it seems as if many routes can lead to similar phenotypes.

During latitudinal range expansion, or as a consequence of climate change, ectothermic organisms can face changes in winter abiotic conditions, such as for instance length of the cold season, its average temperature, the temperature fluctuations or absolute minimum temperature (Bale and Hayward, 2010; Williams et al., 2014). These can act as expansion barriers if the organism cannot cope with the changing conditions, be this through general robustness, phenotypic plasticity or adaptation over generations (Urbanski et al., 2012). One species, that has rapidly expanded its range from relatively benign areas in Northern Mexico into habitats with stronger seasonality, is the invasive CPB, *Leptinotarsa decemlineata* (Say), a major pest of cultivated Solanaceous crops. The capacity of the CPB to overwinter in diapause, where cold tolerance is increased (Boiteau and Coleman, 1996; Lehmann et al., 2015a) and energy consumption decreased through metabolic suppression (May, 1989; Piiroinen et al., 2011; Lehmann et al., 2015b), has been suggested to be of major importance for survival during the invasion into environments with progressively stronger seasonality (Kort, 1990; Alyokhin, 2009; Lehmann, 2013; Izzo et al., 2014). While range expansion in Europe has been rapid (Johnson, 1967), a retardation in expansion speed has been observed during the last 30 years at the northern range margin (EPPO, 2006; Lindström and Lehmann, 2015; Lehmann et al., 2020). This species therefore presents an interesting opportunity to understand factors and mechanisms facilitating and constraining latitudinal range-expansion. Here we compared diapause energetics and temporal changes in lipid composition during diapause in the invasive CPB and two native high-latitude chrysomelids, the MB *Chrysolina polita* (Linnaeus) and the AB *Agelastica alni* (Linnaeus). These are among the closest relatives to the CPB that can be found in the region, since there are no naturally occurring *Leptinotarsa* species in Europe (Silfverberg, 2011). These species overwinter as adults burrowed in the soil (Kort, 1990; Kölsch et al., 2002, Lehmann P, personal observation) and show the typical diapause ecophysiology common to Chrysomelids (Tauber et al., 1986; Hodek, 2012).

We investigate two major ecophysiological stressors during winter; energetic stress and cold stress. Energetic traits include (i) metabolic suppression, (ii) lipid store size, and (iii) lipid store utilization. While it is known that the CPB gathers TAG in its fat body during pre-diapause (Yocum et al., 2011a; Lehmann et al., 2012) it is not known how lipid quantity and quality changes temporally or compares to other high latitude chrysomelid beetles with similar overwintering ecology. For cold stress we study the effects of low temperature on lipid biology, and investigate (iv) HVA in storage and structural lipids. For utilization of lipid stores, even at very low rate under metabolic suppression, storage lipid molecules cannot solidify but have to remain in a semifluid state. This requires modifications in their molecular structure (Raclot et al., 2001; Rozsypal et al., 2014) and we here study average degree of acyl chain unsaturation (expressed as DBI) in TAG species. Similarly, most physiological functions of animals are dependent on the actions of integral membrane proteins, which require specific lipids to surround them (Marèelja, 1976; Wodtke, 1981) and we here study head-group restructurings (seen as changed phospholipid class ratios) and DBI of structural lipids. Lipidome differences in either storage or structural lipids between the invasive CPB and its native relatives related to winter energetics could be indicative of a physiological constraint that is limiting further range expansion, or alternatively tolerance against climate change in the present habitats.

## Materials and Methods

### Study Animals and Rearing Conditions

The CPB were originally collected from fields near Petroskoi in Russian Karelia (61°49′N, 34°10′E) (*N* = 917) in the summer of 2006. These areas represent the current northernmost distribution of the species (EPPO, 2006). In each summer the overwintered CPB adults were maintained under a long-day photoperiod (18 h light, 23°C, 60% relative humidity) to ensure mating. Their offspring were then reared at a short-day photoperiod (12 h light 23°C, 60% relative humidity) and overwintered at 5°C in environmental chambers as described previously (Lehmann et al., 2012). We maintained a large and outbred population over the generations (at least 50 families per generation, over 1000 individuals) to minimize laboratory selection.

For the experiment, which was performed during the summer of 2012, unrelated CPB beetles were mated. Mated pairs were checked for deposited eggs daily. Larvae (10–15 per family) were reared on whole potato plants (which were covered in a plastic housing) in a greenhouse till adulthood at 23°C degrees, 60% relative humidity and 18 h light. The pots were checked daily for emerged adults, which were sexed and weighed (±0.1 mg, AM100; Mettler) and reared under a short-day photoperiod (12 h light). The MB and AB were collected the 14.08.2012 from two locations in central Finland (around lake Jyväsjärvi, 62°22′N, 25°73′E). We tracked the emergence of the summer generation, collected young adults during August and subsequently reared them under short-day photoperiods (12 h light) to induce overwintering. The AB and MB were maintained for 1 month at these conditions before the start of the experiment, to allow them to fully acclimate to the laboratory conditions, and undergo physiological preparation for diapause. A field survey conducted at the collection locations on a monthly basis showed that natural overwintering for the AB and MB was initiated in October (Supplementary Figure S1). Since we collected the beetles very early in the overwintering generation, several months before natural diapause initiation, and maintained them under the same conditions as the CPB in the laboratory, we are confident the diapause initiation program is comparable among the species. All beetles were fed *ad libitum* with fresh leaves, the CPB with potato (*Solanum tuberosum* of the van Gogh variety), the MB with mint (*Mentha arvensis*) and the AB with alder leaves (*Alnus glutinosa* and *Alnus incana*). Beetles were overwintered by decreasing temperature from 23° to 15°, 10° and finally to 5°C with 2 weeks at each intermediate temperature.

Adult beetles were sampled at three time-points during winter. First when the temperature had been lowered from 23°C to 15°C and adults had stopped feeding, but had not yet burrowed into the soil. Importantly, this means the first sampling point reflected when beetles had “chosen” to enter diapause, and thus should be physiologically prepared (Lehmann et al., 2014a, 2015a). The second time-point was 2 months and the third time-point 4 months after they had burrowed to the soil. Beetles were thus acclimated to 15°C in the first time-point and 5°C in the second and third time-point. The time-points were chosen to reflect different diapause phases, the first diapause initiation (0 months), the second diapause maintenance (2 months) and the third the transition between terminated diapause (4 months) and the following low temperature quiescence (Lefevere and De Kort, 1989; Koštál, 2006). Sample sizes for all analyses can be found in Supplementary Table S1.

### Flow-Through Respirometry

Respirometry was performed by measuring gaseous carbon dioxide (CO_2_) production at the three time-points described above. For the first time-point beetles were taken straight from the rearing conditions. For the second and third time-points beetles were first transferred to acclimate at 10°C for 24 h and then dug up from overwintering pots for respirometry. Beetles were kept in darkness throughout the acclimation and measurement process. CPB and MB were measured individually while AB were measured in same-sex pairs. This was done since these beetles primarily showed continuous respiration and the CO_2_ amounts of single beetles generally were below our signal to noise threshold. CO_2_ production was measured for 120 min with a Li-6252 CO_2_ analyzer (LiCor, Lincoln, NE, United States) connected to a flow through respirometry system, as described previously (Lehmann et al., 2014a). Briefly, ambient air was scrubbed free of water vapor (Drierite; WA Hammond Drierite Co Ltd., Xenia, OH, United States) and CO_2_ (Ascarite II; Acros Organics, Fisher Scientific, Pittsburgh, PA, United States) and pumped through the system (SS-2 pump; Sable Systems, Las Vegas, NV, United States) at a flow rate of 150 ml min^–1^ controlled by a mass flow controller (840 Series; Sierra Instruments Inc., Monterey, CA, United States). The respirometry chamber (volume 1.7 ml) was located inside a cabinet (PTC-1; Sable Systems) in which the temperature was set to 15°C and programmed with a temperature controller (Pelt-5; Sable Systems). Preliminary tests were performed to ensure that incurrent air temperature flowing through the respirometry chamber was stabilized with the ambient temperature in the cabinet. The respirometry data were baseline corrected and converted to mL CO_2_ h^–1^ using the acquisition and analysis software ExpeData, version 1.1.15 (Sable Systems). Potential beetle movement was tracked with an infra-red light scattering based activity detector (AD-1; Sable Systems). After the respirometry beetles were weighed (±0.1 mg, AM100; Mettler), and stored whole at −20°C until used for lipid analyses.

### Total Lipid Extraction

Total lipids were extracted using the Folch method (Folch et al., 1957). First, whole frozen beetles were homogenized mechanically in 220 μl milli-Q water in Wheaton homogenizers (low extractable borosilicate glass mortar and PTFE pestle). Then 4.5 ml chloroform:methanol (2:1) was added, and the tubes were vortexed vigorously for 10 s. The homogenate was transferred to Kimax glass tubes and left at 23°C for 30 min in darkness, and centrifuged for 10 min at 3000 rpm to precipitate solid materials. The clear supernatant was moved to a new Kimax tube and 0.9 ml of milli-Q water was added. Following vigorous vortexing (10 s), tubes were again centrifuged for 10 min at 3000 rpm and the lower organic phase was collected to a new tube. This step was repeated on the original tube after substituting the collected lower phase with theoretical lower phase (2.25 ml chloroform/methanol/water 86:14:1). The pooled lower phases of the two extraction steps were vortexed, centrifuged as above, and the clear organic supernatant was moved to a new tube, and evaporated near to dryness under nitrogen stream after which 1 ml of chloroform:methanol (1:2) was added and the sample stored in 1.5 ml chromatography vials (Waters, Milford, MA, United States) for a maximum of 3 days at −20°C until analysis of the lipid class profiles by HPTLC. Aliquots of the extracts were stored at −80°C for mass spectrometric analysis of the detailed lipid species profiles.

### Analysis of Lipid Classes Using High-Performance Thin-Layer Chromatography

Lipid class compositions were analyzed using HPTLC (technical details can be found in Supplementary Table S2). First, silica gel HPTLC 60 F254 plates (Merck, Darmstadt, Germany) were cleaned prior to analyses with chloroform/methanol/acetic acid/water (25:17.5:3.8:1.75) and stored in a desiccator. Samples were removed from the freezer, and dried under nitrogen. Then 500 μl of chloroform: methanol (1:2) was added, the vial vortexed, and samples applied on a silica plate with a Camag Automatic TLC Sampler 4 (Camag, Muttenz, Switzerland). For MB and AB 20 μl of sample was added, and for CPB only 15 μl, as the extract of these larger beetles had higher lipid concentrations. On each plate a range of storage (or neutral) lipid standards of known concentration were also sprayed (Supplementary Table S3). After spraying, the plate was dried for 5 min using a hair dryer. The plate was then placed in a horizontal through chamber (Camag, Horizontal Developing Chamber 2) and the storage lipids were eluted with hexane/diethyl ether/acetic acid/water (26:6:0.4:0.1). For the separation of phospholipids on another plate, samples and standards (Supplementary Table S3) were eluted with chloroform/methanol/acetic acid/water (25:17.5:3.8:1.75). The plate was dried for 5 min using a hairdryer and then developed by first dipping for 6 s (Camag, Chromatogram Immersion Device III) in an aqueous 3% copper sulfate and 8% phosphoric acid, dried for 5 min using a hairdryer, and finally heated in an oven set to 180°C until lipid bands were clearly visible (about 3–4 min). After development, plates were scanned with a Camag TLC plate scanner 3 at 254 nm using a deuterium lamp and with the lipid bands quantified by the Win-CATS 1.1.3.0 software. Resulting chromatographic peaks were identified by comparing their retention times against those of known standards. Then the peak areas were integrated and the amount of lipid per lipid class (in pmol) in the sample counted using the formula:

pmol⁢[sample]=area⁢[sample]÷(area⁢[standard]÷pmol⁢[standard])

The relative concentrations of the lipid classes (molar percentage) were calculated by dividing the pmol content of a specific lipid class with the sum of pmol contents of all major lipid classes detected. In addition, lipid stores are primarily made up of TAG, but also DAG and SE are intrinsic to energy metabolism (Athenstaedt and Daum, 2006; Arrese and Soulages, 2010), and therefore these three lipid classes were summed for calculating total lipid store size in pmol per beetle. For the downstream statistical analyses, the storage lipid contents were corrected for spraying amount differences, transformed to μmol and divided by beetle mass to yield relative storage lipid amount.

### Analysis of Lipid Species Using Electrospray Ionization Mass Spectrometry

Individual lipid species of the main lipid classes were identified and quantified using ESI-MS. Sample extracts were removed from storage and brought to room temperature. Then 10 μl aliquots were dissolved in 60 μl chloroform/methanol 1:2 (by volume) and spiked with 3–6 μl cocktail of internal standards (Supplementary Table S4), the volume adjusted according to the lipid concentration of each sample. The sample solutions were infused into the electrospray source of an ion trap mass spectrometer (Esquire LC, Bruker-Franzen Analytik, Bremen, Germany) and spectra recorded by employing both positive and negative ionization mode in the range of m/z 500–1000 (technical details described in Supplementary Table S5). To confirm the identifications of glycerolipid species, additional ESI-MS/MS precursor or neutral loss scans were recorded for each sample type to verify phospholipid polar head groups (Brügger et al., 1997) and reveal the acyl chain assemblies of TAGs (Murphy et al., 2007) using a triple quadrupole mass spectrometer (Agilent 6490 Triple Quad LC/MS with iFunnel Technology; Agilent Technologies, Santa Clara, CA, United States). The ion trap ESI-MS spectra were processed by Bruker Daltonics (Billerica, MA, United States) data analysis software and the triple quadrupole ESI-MS/MS spectra by Agilent Mass Hunter software. The individual lipid species were quantified by using the internal standards (Supplementary Table S4) and LIMSA software (Haimi et al., 2006) and expressed in mole percentage (mol%). The lipid species were abbreviated as: [chain total carbon number]:[chain total number of double bonds].

Two indexes related to lipid viscosity or packing efficiency in membranes were calculated, the ratio of PE to PC (PE/PC), and average number of double bonds per acyl chain of the lipid species (DBI). The DBI was applied to lipid species to follow the original principle of DBI designed for fatty acids (Kates, 1986) as:

DBI=[∑1×(%monounsaturatedlipidspecies)+2×(%diunsaturatedlipidspecies)…]/[100×(n⁢u⁢m⁢b⁢e⁢r⁢o⁢f⁢a⁢c⁢y⁢l⁢c⁢h⁢a⁢i⁢n⁢s⁢i⁢n⁢l⁢i⁢p⁢i⁢d)]

To facilitate comparisons across different lipids classes with different number of acyl chains (TAG has 3, phospholipids PC, PE, PS and PI all have 2, and SM has only 1 acyl chain in the molecule) the formula reports the double bond contents per single acyl chain (i.e., fatty acid).

### Statistical Analyses

CO_2_ production was used as proxy of metabolic rate, and studied with a generalized linear mixed model using the normal distribution and an identity link. The CO_2_ data was log_10_-transformed due to heavy left-skewness, divided by mass in g and then used as dependent variable. As factorial explanatory variables species (CPB, MB, and AB) and diapause phase (initiation, maintenance, and termination) were added. While preliminary observations suggested beetles moved very little during the measurement, we added the arbitrary activity metric as continuous covariate to correct for slight movement. To investigate how lipid stores varied among the species and diapause phase a generalized linear model with the normal distribution and an identity link was again used. The relative storage lipid amount was used as dependent variable in a model where species and diapause phase were added as factorial explanatory variables. The TAG and membrane lipid viscosity related indexes were analyzed with generalized linear models with the normal distribution and an identity link was again used. Lipid viscosity traits were used as dependent variable in individual models and species and diapause phase as factorial explanatory variables. Non-significant interactions were removed from the final model (Sokal and Rohlf, 2003), whose improvement was also tracked using the Akaike Information Criterion. For significant interactions, the main level effects were *post hoc* tested with univariate F-tests and pair-wise tests on group estimated marginal means with Bonferroni multiple-comparison corrections. Significant main effects were subjected to the same pairwise testing procedure. For analyzing CO_2_ production, storage lipid content and viscosity indexes the IBM SPSS statistics 25.0 (IBM SPSS Inc., Chicago, IL, United States) statistical software package was used. Finally, for overall description of lipid species dynamics, the molar percentages of individual lipid species were divided by 100, ArcSin transformed, block-normalized and standardized, after which they were subjected to PCA (Sirius 8.0, Pattern Recognition Systems AS). The last step involved forcing standard deviations to be equal, while allowing means to fluctuate. Thus, the common lipid species do not dominate the patterns, potentially obscuring important changes in relative abundance of less common lipid species. Statistical differences among groups in main components were investigated using SIMCA (Wold and Sjöström, 1977) with an alpha level of 0.05. The PCA analysis and subsequent SIMCA was run separately for storage and phospholipid species.

## Results

### Metabolic Suppression

**FIGURE 1:**
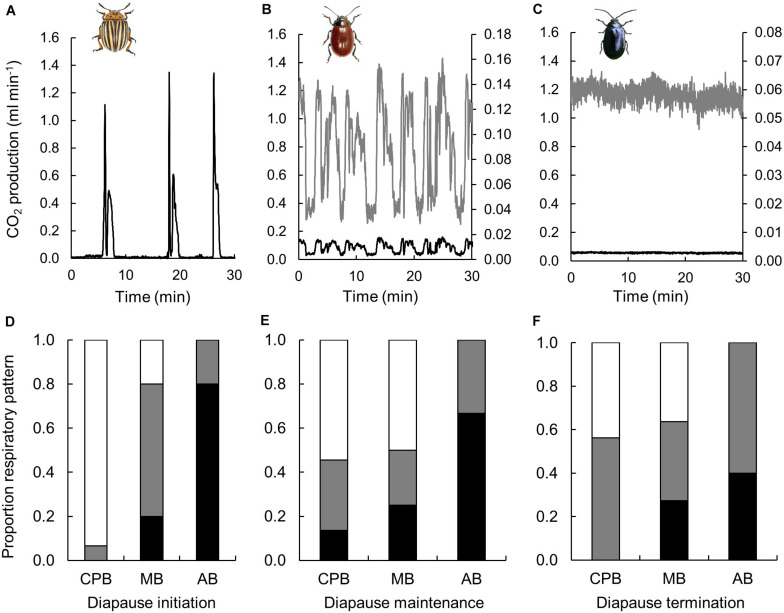
Respiratory patterns varied during winter in three high-latitude chrysomelid beetle species. Panels **(A–C)** show examples of discontinuous gas exchange in a CPB **(A)**, of cyclic gas exchange in a MB **(B)** and continuous gas exchange in an AB **(C)**. The black line shows CO_2_ production on an equal scale, while the gray trace in **(B,C)** shows the same traces scaled for clarity (secondary *y*-axis). These traces are all from stationary individuals, of which a sample image is shown in the inserts. Panels **(D–F)** show proportions of CPB, MB and AB showing discontinuous (white), cyclic (gray) or continuous (black) gas exchange during diapause initiation **(D)**, diapause maintenance **(E)** and diapause termination **(F)**. Insert beetle figures by (a) U. Schmidt, (b) www.ukbeetles.co.uk, and (c) Trevor and Dilys Pendleton/www.eakringbirds.com.

Three diapause phases were investigated, of which the first (0 months) represented diapause initiation still at relatively high temperature (15°C), and the subsequent represented diapause maintenance (2 months) and diapause termination (4 months) at a lower temperature (5°C). Note that the measurements were performed at a standardized temperature of 15°C. Respiratory patterns varied dramatically among species (Figure 1). The CPB displayed cyclic and DGC and only very few cases of continuous gas exchange. The MB displayed all the three respiratory patterns, and the AB only displayed continuous or cyclic respiration. For the CPB the proportion of DGC decreased as diapause progressed. In the MB the proportion displaying continuous gas exchange was quite static (around 0.2) but the proportion showing DGC increased with time in diapause. The proportion of AB displaying cyclic gas exchange increased as diapause progressed.

**FIGURE 2:**
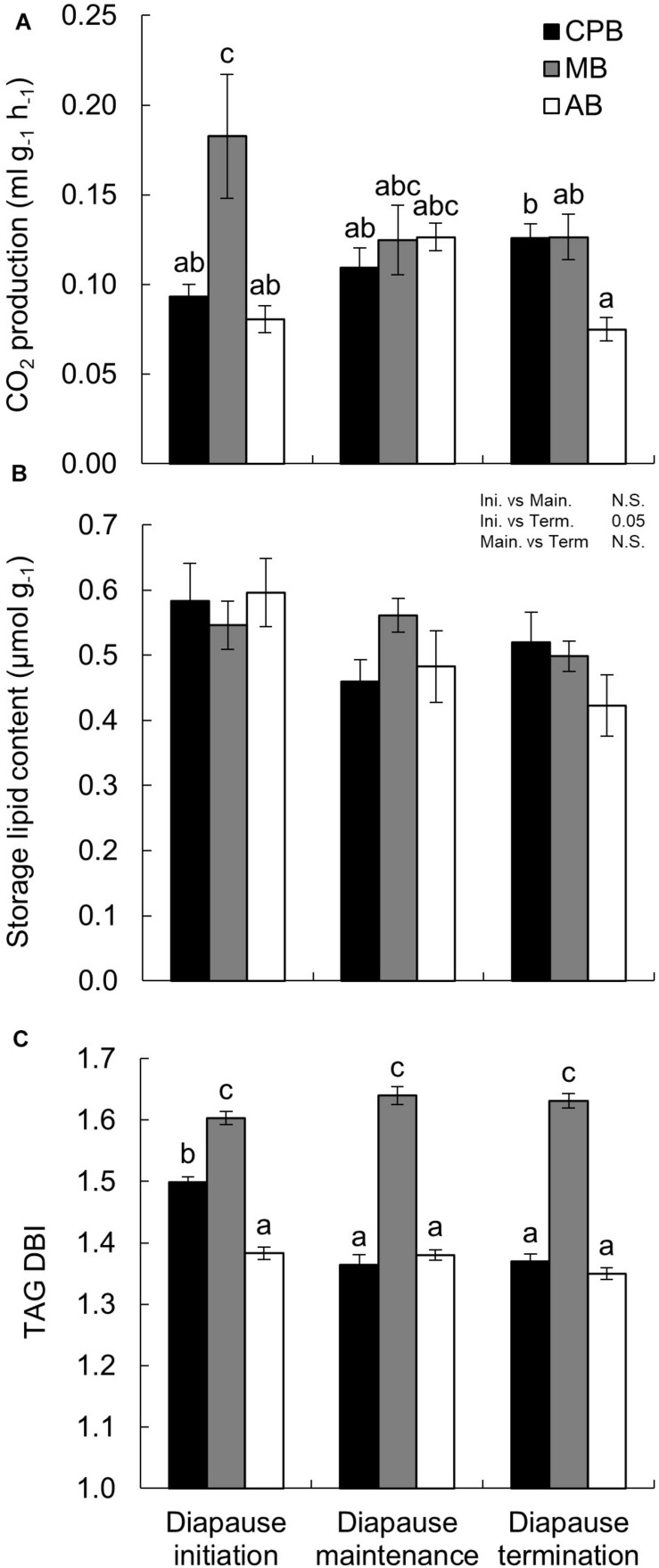
Winter energetics and phospholipid viscosity in three high-latitude chrysomelid beetle species. **(A)** CO_2_ production as a function of diapause phase. Note that while mass-specific CO_2_ production is shown on the *y*-axis, the statistical model used mass as covariate. **(B)** Mass-corrected total storage lipid content as a function of time in diapause. **(C)** Double bond index (DBI) of triacylglycerol (TAG) calculated per acyl chain (three-fold for intact TAG molecule). Bonferroni corrected pairwise comparisons are shown in each panel. Comparisons between main effects are shown above the panel. **(A,C)** Letters show comparisons for the interaction between main effects. No common letters denotes *P* < 0.05. All numbers are expressed as mean ± standard error of mean.

Additional signs of metabolic suppression were investigated by measuring the CO_2_ production of the beetles. Overall, the three species showed relatively similar average CO_2_ production values (Table 1A), especially at later diapause phases. However, CO_2_ production profiles differed between the species. The CPB showed a consistent increase in CO_2_ production as diapause progressed. In contrast, the MB showed a consistent decrease in CO_2_ production. In the AB, the CO_2_ production first increased between diapause initiation and maintenance, and then decreased as beetles transitioned from diapause maintenance to termination (Figure 2A). While the difference for both the CPB and the MB between diapause initiation and termination were significant, overall absolute differences among species within diapause phases were relatively minor.

**TABLE 1:** Final generalized linear models describing how **(A)** CO_2_ production and **(B)** storage lipid content changed during diapause in three high-latitude Chrysomelid beetles.

Effect	Wald chi-square	df	Significance
**(A) CO_2_ production**			
Intercept	1.521	1	0.217
Activity	6.534	1	0.011
Species	13.941	2	0.001
Diapause phase	4.585	2	0.101
Species × diapause phase	21.225	4	<0.001
**(B) Total storage lipid content**			
Intercept	1260.200	1	<0.001
Species	0.730	2	0.694
Diapause phase	8.098	2	0.017

### Lipid Classes and Overall Lipid Species Composition

The quantitatively most important structural lipid classes of membranes were PC, SM, PE, PS+PI, and free sterols, including cholesterol (Ster) (Supplementary Figure S2). The quantitatively most important storage lipids were TAG, DAG, and SE (Supplementary Figure S2). In all beetle species TAGs dominated the lipid profile and made up 50–60% of the total lipid present. Removing the storage lipid classes revealed underlying dynamics in structural lipid classes that otherwise was obscured. The SM, PS+PI and Ster showed variation with time in diapause. These changes were, however, limited to the MB and AB (Supplementary Table S6). In the CPB, no significant changes occurred in any structural lipid class as a function of time in diapause, and there was overall much variation among individuals.

**FIGURE 3:**
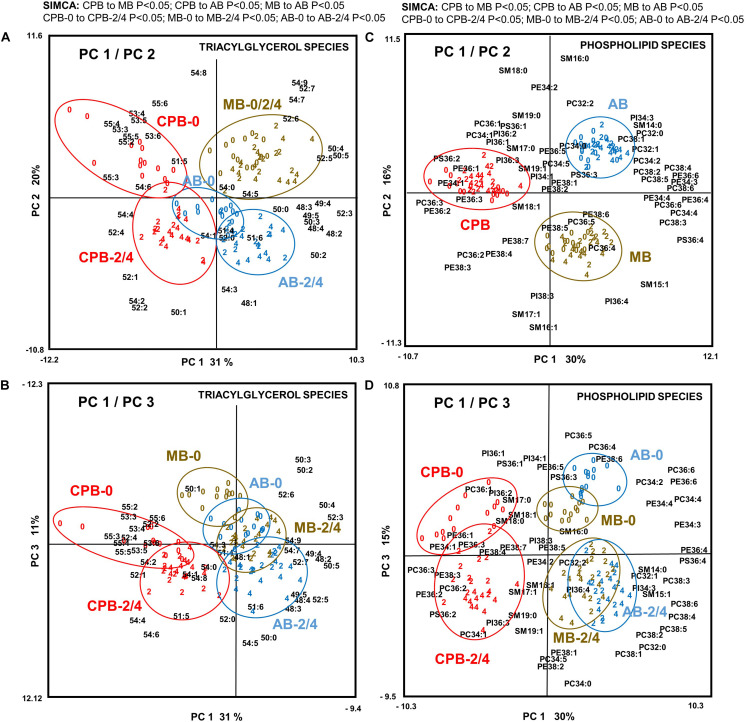
Principal component analysis of all **(A,B)** 42 triacylglycerol (TAG) species and **(C,D)** 58 phospholipid species in the three investigated beetles (*N* = 114) showing **(A,C)** PC1 and PC2 and **(B,D)** PC1 and PC3. For TAG species, these three axes explained 31, 20, and 11% of variation, respectively (i.e., over 60% together). Plotting the data with the coordinates PC1 and PC3 gave further separation related to the phase of the diapause. For phospholipid species, these three axes explained 30, 16, and 15% of variation, respectively (i.e., over 60% together). The data consisted of relative molar percentage (%) values for each lipid species within its lipid class, which was ArcSin-transformed and Block-normalized before analysis. Beetles have been color-coded so that red symbols represent CPB, brown are MB, and blue are AB individuals. Individual beetles are marked by a number referring to diapause phase (0 = diapause initiation, 2 = diapause maintenance at 2-month time-point, 4 = diapause termination at 4-month time-point). The results of pair-wise tests using soft independent modeling of class analogies (SIMCA) (*P* < 0.05) are listed on top of panels **A** and **C**.

The PCA of TAG species profiles showed two main axes of variation that together explained 51% of the variation in TAG data (Figure 3A). PC1 (explaining 31% of variation) likely reflects the difference between the invading species and the two native species, while PC2 (explaining 20% of variation) separates both the MB from the AB, and all diapause initiation samples of CPB and AB from their later diapause phases. PC3 (explaining 11% of variation) is required to separate the diapause initiation samples of the MB from the later diapause phases in that species (Figure 3B). SIMCA comparisons of two sample groups at a time (above Figure 3A) showed that in all the three beetle species the TAG lipid species composition shifts dramatically from diapause initiation toward diapause maintenance but then remains relatively similar until termination (above Figure 3A). The individual TAG species affecting the separation most strongly are the highly unsaturated species, enriched in initiation samples, and several species with 0–2 double bonds, which associate with the later phases of diapause (Figure 3). The detailed TAG species profile was largely changed in all three beetle species (Figure 3 and Supplementary Figure S3).

The PCA of phospholipid species profiles showed three main axes of variation that together explained 61% of variation in phospholipid data (Figure 3C). PC1 (explaining 30% of variation) likely reflects the difference between the invading species and the two native species, while PC2 (explaining 16% of variation) seems to separate the MB from both the AB and CPB, and finally PC3 (explaining 15% of variation) seems to describe the progression from early diapause to later diapause phases. The phospholipid species with highest loadings for PC1 are either polyunsaturated species, which were found in higher amounts in the AB and MB, or those with 1–3 double bonds, which were more common in CPB that in the other beetle species. The separation along PC2 was largely driven by different degree of unsaturation of SM species; more monounsaturated SM species in MB and more saturated SM species in the other beetle species. For PC3, the major drivers seem to be the acidic PI and PS species, and highly unsaturated PC and PE species, all promoting cellular metabolism, and are associated with diapause initiation (Figure 3D). Further, the PC3 axis demonstrates that in later diapause phases the overall degree of unsaturation seems to decrease and the relative proportions of several SM species increase.

The species profiles were also compared separately in each lipid class. Such comparisons in one diapause phase at a time resulted in statistically significantly different profiles for all the beetle species pairs and all the major lipid classes TAG, PC, SM, and PE (as the only exception, MB and AB had similar PC profiles at the initiation phase of diapause). In general, the beetle species comparisons at certain time points resulted statistically significant differences more often than the time point comparisons in one species (Table 2). The class specific lipid species profiles that were tested are also shown as bar-plots (Supplementary Figures S3–S8).

**TABLE 2:** Statistically significantly different species profiles when each lipid class was studied separately during the initiation, maintenance and termination phase of diapause in three high latitude Chrysomelid beetles.

(A)	Diapause initiation (0)			Diapause maintenance (2)			Diapause termination (4)		
	CPB-MB	CPB-AB	MB-AB	CPB-MB	CPB-AB	MB-AB	CPB-MB	CPB-AB	MB-AB
TAG	*	*	*	*	*	*	*	*	*
PC	*	*	*	*	*	*	*	*	*
SM	*	*	*	*	*	*	*	*	*
PE	*	*	*	*	*	*	*	*	*
PS	NS	NS	NS	*	NS	NS	*	NS	NS
PI	NS	*	*	*	*	*	*	*	*

**(B)**	**CPB**			**MB**			**AB**		
	**0–2**	**0–4**	**2–4**	**0–2**	**0–4**	**2–4**	**0–2**	**0–4**	**2–4**

TAG	*	*	NS	*	*	NS	*	*	NS
PC	NS	*	NS	*	*	NS	*	*	NS
SM	*	NS	NS	NS	NS	NS	*	*	NS
PE	NS	NS	NS	*	*	NS	*	*	NS
PS	NS	NS	NS	NS	NS	NS	NS	NS	NS
PI	NS	NS	NS	NS	NS	NS	*	NS	NS

Displayed are pairwise comparisons using SIMCA, where *means *P* < 0.05 for the specific comparison, and NS means a non-significant result. **(A)** shows differences among species within diapause time-points, while **(B)** shows differences within species, among time-points (0 = diapause initiation, 2 = diapause maintenance, 4 = diapause termination). The species profiles in each lipid class and time-point of the beetle species are presented in Supplementary Figures S3–S8. TAG, triacylglycerol; PC, phosphatidylcholine; SM, sphingomyelin; PE, phosphatidylethanolamine; PS, phosphatidylserine; PI, phosphatidylinositol.

### Lipid Store Size and Utilization

Storage lipid content was analyzed as the sum of TAG+DAG+SE (together amounting to over 70% of lipid present). The relative lipid amount accumulated before diapause was very similar in the three species (Figure 2B) and did not differ significantly between the species in any of the diapause phases (Table 1B). In all species, however, did lipid stores decrease as diapause progressed (Figure 2B), non-significantly between diapause initiation and maintenance, but significantly between these two phases and diapause termination (Figure 2B and Table 1B).

### Homeoviscous Adaptation in Storage Lipids

While the three species shared an overall decrease in lipid stores size, the composition and viscosity of the main storage lipid, TAG, differed in a more complex manner. The CPB had a moderate TAG DBI which decreased as diapause progressed and then stabilized (Figure 2C and Table 3A). The MB had an overall high TAG DBI which did not change during diapause. The AB had an overall low TAG DBI which did not change during diapause.

**TABLE 3:** Final generalized linear models describing how **(A)** TAG DBI, **(B)** PE/PC ratio and **(C–G)** DBI of major phospholipid classes changed during diapause in three high-latitude Chrysomelid beetles.

Effect	Wald chi-square	df	Significance
**(A) TAG DBI**			
Intercept	166481.843	1	<0.001
Species	960.600	2	<0.001
Diapause phase	29.021	2	<0.001
Species × diapause phase	88.677	4	<0.001
**(B) PE/PC ratio**			
Intercept	6020.654	1	<0.001
Species	370.862	2	<0.001
Diapause phase	0.866	2	0.649
**(C) PC DBI**			
Intercept	22957.731	1	<0.001
Species	68.801	2	<0.001
Diapause phase	20.402	2	<0.001
**(D) SM DBI**			
Intercept	24047.125	1	<0.001
Species	1625.385	2	<0.001
Diapause phase	2.280	2	0.320
**(E) PE DBI**			
Intercept	119853.795	1	<0.001
Species	296.708	2	<0.001
Diapause phase	1.874	2	0.392
Species × diapause phase	13.253	4	0.010
**(F) PS DBI**			
Intercept	141945.875	1	<0.001
Species	289.818	2	<0.001
Diapause phase	1.150	2	0.563
**(G) PI DBI**			
Intercept	57383.026	1	<0.001
Species	58.653	2	<0.001
Diapause phase	21.840	2	<0.001

### Homeoviscous Adaptation in Structural Lipids

**FIGURE 4:**
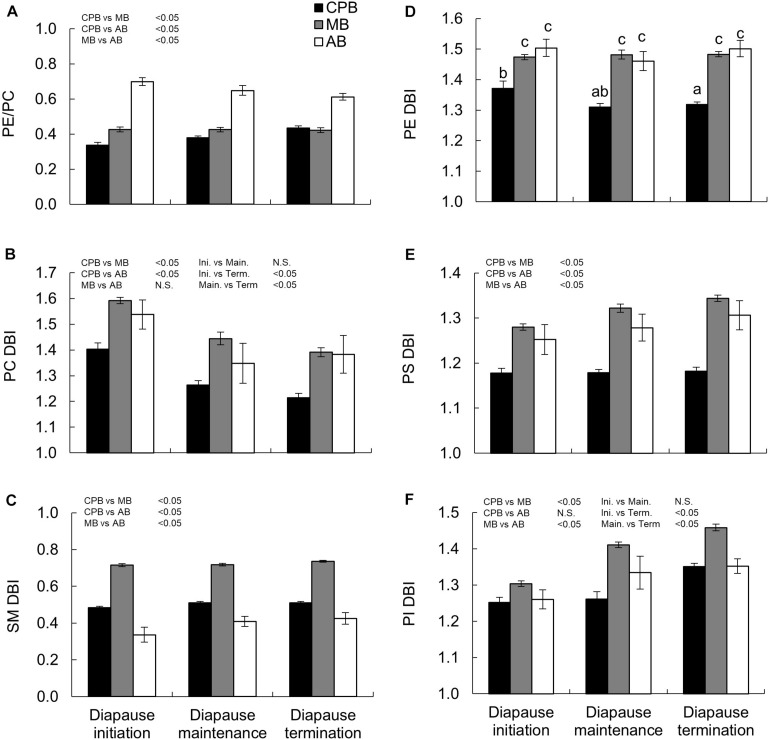
Winter energetics and phospholipid viscosity in three high-latitude chrysomelid beetle species. **(A)** Ratio of PE to PC phospholipids, **(B–F)** unsaturation indexes (double bond index, DBI) calculated for all phospholipid classes studied, and expressed per acyl chain for easy comparison between lipids classes with 1 (SM), 2 (PC, PE, PS, PI), or 3 (TAG in Figure 2C) acyl chains. Bonferroni corrected pairwise comparisons are shown in each panel. Comparisons between main effects are shown above the panel. For **(D)** letters show comparisons for the interaction between main effects. No common letters denotes *P* < 0.05. All numbers are expressed as mean ± standard error of mean. Lipid abbreviations are: PE, phosphatidylethanolamine; PC, phosphatidylcholine; SM, sphingomyelin; PS, phosphatidylserine; PI, phosphatidylinositol; TAG, triacylglycerol. For calculation of PE/PC ratio, the diacyl species of PE and PC (no ether species) were used.

Two metrics of phospholipid viscosity were calculated, the ratio of PE to PC and the unsaturation degree of fatty acids in the main phospholipids (PC, SM, PI, PS, and PE), expressed as DBI. For easy comparison the DBI values were calculated per acyl chain, despite that the lipids studied have 1–3 acyl chains in the molecule. There were several clear differences among species and diapause phases in these indexes (Table 3), and since the species × diapause phase interactions was significant only for PE DBI, the models suggested relatively straightforward shifts for most of the traits. The PE/PC ratio was highest in the AB, similar in the MB and CPB and did not change statistically significantly with diapause phase in any beetle species (Figure 4A). Still, the AB had slight decreasing and CPB slight increasing tendency in their PE/PC ratio as diapause progressed. The PC DBI was lower in the CPB than the two native beetles, and decreased significantly as diapause progressed in all species (Figure 4B) albeit the effects were relatively minor. The SM DBI stayed relatively unchanged during diapause, and when the beetle species are compared, the MB had a much higher SM DBI than the other two species, which did not differ from each other (Figure 4C). The PE DBI was high and stable in both the MB and AB during diapause, while the CPB displayed both a lower overall PE DBI, which also decreased with time in diapause (Figure 4D). The PS showed similar DBI patterns as PE with little dynamics during diapause, and the AB and MB having an overall higher degrees of DBI than the CPB (Figure 4E). The PI DBI increased significantly as diapause progressed in all species, and also differed among the species so that the MB again had the overall highest DBI (Figure 4F).

## Discussion

The present study investigates how a recently invaded beetle compares to two native beetle species, from the same latitudinal origin, in several traits related to two key stressors that insects need to overcome during diapause, energetic stress and cold stress. Failure to cope with these could lead to mortality or otherwise reduced fitness and act as an expansion barrier for species shifting range into habitats with harsher winters. Understanding how native beetles cope with these challenges, and characterizing their physiological toolbox, can therefore provide valuable insight into the mechanisms behind high-latitude seasonal adaptations. Overall, the data suggest that the invasive species is comparable or even superior to the native beetles in terms of managing energetic stress, but shows an overall more varied and less canalized diapause lipidome, suggesting less evolved capacity to manage cold stress. This latter finding is in agreement with findings of a previous study of the same three beetles, that showed the invasive species had an overall poorer tolerance against direct cold shocks, and less well canalized metabolome, than the native species (Lehmann et al., 2015a). The main findings are summarized in Table 4 and discussed in detail below, in a trait-by-trait manner. Together these studies suggest that the CPB shows a diapause phenotype less well adapted to high-latitude conditions than native relatives, which might potentially be one reason for the retardation of expansion to higher latitudes observed globally over the last decades. It is even possible that the buffering effect of their microhabitats has constrained selection to act on diapause-related physiology and allowed the CPB to spread into harsh winter climates primarily due to its behavioral plasticity (Izzo et al., 2014; Lehmann et al., 2014a). It would therefore be important to systematically investigate the influence of overwintering microhabitat and field conditions (Yocum et al., 2011b) on energy fluxes and cold tolerance in the three species in future experiments. Also, studies should compare these traits among populations, to see if selection already is acting on these traits within the European range of the CPB (Lyytinen et al., 2012; Lehmann et al., 2015c). It should also be noted that even though we maintained a large and outbred laboratory population of the CPB, and have no reason to suspect laboratory selection and drift, a future study should compare the species when collected straight from the field, or after being kept for similar periods in the laboratory for more direct comparison.

**TABLE 4:** Summary of the results related to oxidative energy metabolism and membrane properties during different phases of diapause in three high-latitude Chrysomelid beetles, including potential implications of results with regard to further range expansion and climate change in local overwintering habitats.

	CPB	MB	AB
**Oxidative energy metabolism traits:**			
Gas exchange pattern	Discontinuous	Cycling	Continuous
Metabolic suppression	High	High	High
Relative lipid store size	Large	Large	Large
Lipid store utilization	Stable → slight decrease	Stable → slight decrease	Decrease
Storage TAG unsaturation (DBI)	Average, decrease	High, no change	Average, no change
**Membrane properties:**			
PE/PC ratio and its change (head-group restructuring)	Low, little change	Average, no change	High, little change
PC unsaturation (DBI)	Average, decrease	High, decrease	High, decrease
SM unsaturation (DBI)	Average, no change	High, no change	Low, no change
PE unsaturation (DBI)	Average, slight decrease	High, no change	High, no change
PS+PI (acidic phospholipid classes) unsaturation (DBI)	Low, increase in PI	Average → high, increase	Average → high, increase
**Possible implications:**			
Range expansion	Need for profound restructuring of membrane lipids may limit northward expansion.	Wide current range reflected by strong winter energetic and cold stress adaptations.	Adapted to high latitude conditions, expanding south not likely.
Climate change	Discontinuous respiration and stable lipid stores indicate buffering capacity against warming winters. Further, milder winter temperatures could require less membrane restructuring, thus facilitating expansion.	Cyclic respiration and stable lipid stores suggest buffering capacity against warming winters exist.	Continuous respiration and decreasing lipid stores suggests poor capacity to handle warming winters.

*Note that the degree of unsaturation in each lipid class refers to the average double bond content per acyl chain, and even if the average degree of unsaturation is not changed, numerous alterations in the proportions of individual lipid species may occur.*

### Metabolic Suppression

The three beetle species investigated in the present study all showed signs of suppressed metabolism. Comparing CO_2_ production values of CPB during diapause initiation (i.e., at 15°C) to non-diapause individuals (Lehmann et al., 2014a) suggests that CO_2_ production, and thus metabolic rate, is suppressed to about 10% of non-diapause values. The extent of suppression is more difficult to assess for AB and MB as no beetles have been measured during summer using a similar setup. For AB, metabolism appears fully reversibly suppressed to about 5% of normal resting metabolic rate when they are subjected to acute hypoxia (Kölsch et al., 2002), which might be a naturally occurring condition during diapause (Große and Schröder, 1984). These numbers reflect what has been reported in other insects (Williams et al., 2015; Lehmann et al., 2016), and suggest metabolic suppression to be a good phenotypic marker of diapause maintenance in these species. Overall, the results suggest that the invasive CPB has similar or even stronger capacity to suppress metabolism, when compared to the native species.

The beetle species showed large differences in respiratory patterns. For CPB and MB, most individuals, especially in the later diapause phases, showed DGC. This is in general agreement with previous data on diapausing insects, including for instance beetles (Lehmann et al., 2015b; Ploomi et al., 2018), bumblebees (Beekman and Stratum, 1999), stinkbugs (Ciancio, 2018) and butterflies (Schneiderman and Williams, 1953; Jõgar et al., 2004; Stålhandske et al., 2015). It is commonly seen during periods of stress or during periods of suppressed metabolism (Lighton, 2008). Since diapause constitutes both, DGC would be expected, especially given that the three investigated species all spend winter burrowed into the soil, thus potentially facing hypercapnic environments. DGC as protective mechanism against hypercapnia has been suggested as one putative reason why it has evolved, and why it is common in fossorial insects (Lighton, 1998). Since the theory is relatively clear about the benefits entailed by DGC (Chown et al., 2006; Matthews and White, 2011), it is interesting why the AB did not display any DGC. One potential explanation is that the AB has a different overwintering microhabitat, in wet soil, and thus could display a different eco-physiological strategy than the MB and CPB (but see Hodek, 2012). This environment may be water-loaded and thus potentially frozen (Große and Schröder, 1984; Kölsch et al., 2002; Lehmann et al., 2015a). If the AB overwinter in frozen microhabitats then metabolic restrictions should be different. First, moist and frozen soils are expected to have higher thermal conductivity than dry soils (Lal, 2017), and therefore the AB might experience overall colder overwintering conditions than the MB and CPB. If this is the case, then metabolism should be inherently more suppressed as a consequence of lower ambient temperature. Further, water-loaded or frozen soils might become hypoxic to a higher degree than dry soils and thus AB would face oxygen limitation that likely would act to suppress metabolism (Guppy and Wither, 1999). While it is tempting to suggest that overwintering in frozen microhabitats might indicate that the AB themselves might be frozen (which should act to heavily suppress metabolism), previous studies on the AB suggest this is not the case, even partially (Lehmann et al., 2015a). It can be noted that the CPB prefers to overwinter in porous soils with medium water loading, if given a choice, and this behavior might be explained to some degree by hypoxia avoidance (Ushatinskaya, 1977; Noronha and Cloutier, 1998). Monitoring the abiotic conditions in actual overwintering microhabitats of the AB and MB would be important in future studies.

### Lipid Store Size, Utilization and Homeoviscous Adaptation of Storage Lipids

The three species gathered large lipid stores prior to winter. The proportion of TAG, which represents the main storage form of lipid, was about 50–60% of total lipid in all species. The amount stored per unit of body mass was very similar in the three species during diapause initiation, and while the interaction effect between species and diapause was non-significant, some differences between the species seem likely. While there was an overall decrease in all species between the early and later phases in diapause, an analysis on the data split by species (not shown) suggests that the effect of diapause phase is only significant in the AB, which thus seems contribute the most to the overall decrease in lipid stores. This could again be related to the overwintering microhabitat of the AB. If it normally experiences lower winter temperatures than the other species, it might be less-well equipped for handling the relatively high diapause maintenance temperatures used here (5°C) (e.g., due to lack of DGC). Such a pattern would coincide with the much higher cold tolerance found in the AB compared to the other two species previously (Lehmann et al., 2015a). In any case, the results suggest that the invasive CPB gathers similar amounts of lipid and utilizes them at a rate comparable to, or even more economical than, the two native beetle species.

With progressing diapause, the PCA of TAG species in the three species, but especially the CPB, showed a strong decrease in the relative abundance of TAGs containing PUFAs, a response mirrored by the decrease in TAG DBI in the CPB. In mammals it has been shown that TAG species harboring polyunsaturated acyl chains are most rapidly hydrolyzed, due to their higher hydrophilicity, which exposes them to cytosolic lipases (Raclot, 2003). Thus, even though total storage lipids only show a moderate decrease, these findings lend support to the notion of lipid oxidization for energy production during diapause, especially in the CPB. While lipids likely are used for energy production in all three species, overall rate of lipid consumption was low (in terms of decreasing storage lipid size). This is reflective of patterns found also in other studies (Irwin and Lee, 2003; Williams et al., 2012; Fründ et al., 2013; Lehmann et al., 2016) and probably highlights the effect of metabolic suppression (Sinclair, 2015). It is of course also possible that other substrates are used for energy production than lipid, even though lipid seems most commonly used (Hahn and Denlinger, 2007; Toprak et al., 2020). In the CPB it has for instance been shown that large quantities of a storage protein called Diapause Protein 1 is sequestered prior to diapause (Lehmann et al., 2014b). While primarily consumed after diapause (Lefevere et al., 1989; De Kort and Koopmanschap, 1994) it could provide energy throughout diapause at low rate. It would therefore be important in future studies to directly assess lipid and energy utilization in a more direct manner, by for instance measuring respiratory quotient (RQ) (Schmidt-Nielsen, 1990) or using metabolic tracers (Williams et al., 2016), in order to more firmly establish energy substrate. Finally we note that the MB showed a much higher TAG DBI than the other two species from the outset. This could be due to the very high 18:3n-3 contents of mint leaves (Rao and Lakshminarayana, 1988; Xu et al., 2020) consumed by the MB, which likely explained the high proportions of the TAG species 54:9 (3 × 18:3) and consequently, the high overall TAG DBI.

### Homeoviscous Adaptation of Structural Lipids

For cold stress, the present paper investigates a core mechanism of membrane homeostasis – HVA (Sinensky, 1974; Lewis et al., 1989; Hazel, 1995), and finds results that support it, and details that seem to refute it. Most importantly, the adaptive modifications of lipids were clearly species-specific, and did not follow one simple “beetle pattern.” For lipid classes, two results that refuted generally expected HVA responses stood out. In no species was there a marked increase in the molar ratio of PE to PC when they entered diapause. An increase in this ratio would be expected, since it would lead to impaired molecular packing and thus increased membrane fluidity (Šlachta et al., 2002; Koštál, 2010; Rozsypal et al., 2013). Thus, this mechanism does not seem to be employed in the seasonal acclimatization process of the studied beetle species but apparently played a role in their evolutionary temperature adaptation. As a sign of this, the AB had a relatively high baseline PE/PC ratio, in line with the fact that this species overwinters in the coldest microhabitats of the three species (close to the surface, potentially close to frozen water) (Hiiesaar et al., 2018). However, the present experiments were performed under common-garden conditions, and thus it is unclear whether the species would have shown similar responses if acclimated to natural overwintering microhabitats. Another result refuting the HVA is the general increase in molar percentage of SM, as an increase in this lipid class is expected to increase structural integrity in lipid membranes. Still, as will be described below, there were changes in the lipid species composition of SM that do lend support to HVA. It would be interesting to further explore even lower temperatures than 5°C, since this temperature, at least for the AB, is higher than what the beetles normally face. Indeed, we might partly be observing high-temperature-related stress responses in this species.

Overall, the changes in the phospholipid species profiles found between the different diapause phases were complex, and the differences nuanced between the three Chrysomelid beetles. Indeed, the three species showed distinct phospholipid species profiles, albeit that the AB and MB grouped closer together on the PC1-axis than the CPB. The null-hypothesis in the current experiment was that phospholipid species composition would show little dynamics during diapause. This would indicate that the lipidome is formed prior to winter and then kept static during diapause (Lehmann et al., 2016), which could be explained by metabolic and biochemical costs of membrane remodeling during low-temperature and potentially hypoxic conditions (Bhat and Block, 1992). Since all three species showed large dynamism during the transition from diapause initiation to diapause maintenance, the original null-hypothesis could be rejected. This dynamism then decreased, because only small differences in the later diapause phases were observed. Since the transition from diapause initiation to diapause maintenance in the current experiment is associated with a temperature decrease from 15 to 5°C, the data strongly suggest that we are observing a plastic temperature-dependent acclimation response, even though other factors, including endogenous diapause development, are likely also important. These data therefore do lend support to the notion of a relatively static lipidome during diapause maintenance (Koštál et al., 2013; Lehmann et al., 2016), putting it in contrast to the dynamism seen in the metabolome and transcriptome of these and other species during the maintenance phase (Lehmann et al., 2015a, 2018; Ragland et al., 2015; Koštál et al., 2017).

The main qualitative change in phospholipid dynamics as beetles transitioned from diapause initiation to maintenance in all species was a shift of PC species profile toward a higher diversity in the length of acyl chains. After entering diapause, in this lipid class, the species with short (32- and 34-carbon) and long (38-carbon) acyl chains were favored, at the expense of the species with mid-length (36-carbon) acyl chains. These data suggest that the PC species profile becomes more complex as diapause progresses. The confirmatory fatty acid analyses conducted for each of the species by gas chromatography indicated that the lipids included only six quantitatively important fatty acids: 16:0, 16:1n-7, 18:0, 18:1n-9, 18:2n-6 and 18:3n-3, and several minor ones. Thus the pairs for 32-carbon phospholipid species are mainly comprised of two 16-carbon fatty acids (but see Lehmann et al., 2012). For 34-carbon species, the pair consisted of 16- and 18-carbon fatty acids, while for the lesser 38-carbon species the pair consisted of 18- and 20-carbon fatty acids. The change to a more diverse fatty acid chain length composition in lipid species has been suggested by Lewis et al. to be one hallmark of DPB (Lewis et al., 1989). In their study, they were focused on PE- species, but in our study the phenomenon was clearer for PC- species. For PE species, all beetle species showed relatively little dynamics related to the phase of the diapause, but still differed in their compositions. Both the MB and AB had a large proportion of unsaturated lipid species already during diapause initiation (the PC species 36:4, 36:5 and 36:6, and the PE species 36:5, 36:6 and 38:6). Arranging the beetle species according to their overall degree of PE unsaturation showed that the CPB had clearly lower double bond content of PE than the MB and AB, congruent with HVA-expectations. Later in diapause, the increase in long PE-species (PE 38:1 and PE 38:2) likely enhanced the DPB influence in all the species. Although PE DBI seems to be set high in MB and AB and low in CPB, the PE profile in all three species reacted little to the progression of diapause, in fact, the only temporal trend was a slight decrease in the PE DBI for the CPB, bringing it even further from that of the other species.

A feature that was common to all three beetle species was a decrease in the proportions of PS+PI between diapause initiation and the later diapause phases. These decreases support the idea of regulated metabolic suppression since both PI and PS with their derivatives have important roles in metabolism in the inner leaflets of biological membranes (Corbalán-García and Gómez-Fernández, 2014; Kay and Fairn, 2019). Apparently, the membrane lipidome changes contribute to down-regulation of metabolism. Comparing the PS and PI species profiles between diapause initiation and especially diapause termination further shows that unsaturation overall is increasing in these lipid classes, when PI species that primarily are monounsaturated early in diapause, become polyunsaturated later (Supplementary Figures S7, S8). This finding is in clear agreement with the expectations of the HVA-hypothesis but at the same time the polyunsaturated species are the preferred precursors for the production of signaling derivatives (Vrablik and Watts, 2013). For SM species the data suggested interesting differences between the species. First, the AB had an overall low SM DBI. Increasing the DBI of SM occurs primarily through conversion of saturated to monounsaturated fatty acids via transcriptionally activated Δ9-desaturase (Ballweg and Ernst, 2017). Since this process is oxygen dependent (Bai et al., 2015) the AB, which might face periods in hypoxia or even anoxia during diapause, might be challenged to achieve unsaturation this way, and might instead be more dependent on dietary PUFA-containing lipids. In the CPB, which seems more aerobic during diapause, there was a small increase in the DBI of this lipid class as winter progressed, while the MB instead had a high SM DBI from the outset.

## Conclusion

Overall, our results show that the invasive CPB has a powerful capacity of metabolic suppression and while it utilizes part of its lipid stores during diapause, as do the native cold-adapted MB and AB, lipid stores diminish only modestly. As pertains to the original hypotheses, for the energetic traits investigated, the invasive CPB shows comparable or better capacity for energy management during diapause, than the native species. However, since the CPB has a less canalized diapause lipidome, with overall large inter-individual variability than the native species, the current study suggests a diapause phenotype that is less well adapted to high-latitude conditions than native relatives. This could potentially be one reason for the retardation of expansion to higher latitudes observed globally over the last decades. Still, the data presented here shows that several relevant energy metabolism and cold-tolerance related mechanism are in place and likely could be targets for future selection also in the CPB, and further studies should target different populations across the CPB-range to see whether selection already is acting to improve cold tolerance in this important pest species. At least the metabolic diversity of the CPB, larger than what was detected in the MB and AB, could suggest capacity for evolutionary adaptation through natural selection. With warmer future winters, and provided that the phenotypes of the native MB and AB show little plasticity, they even risk facing metabolic and thermal stress during winter if diapause is shortened, food resources do not increase, and the biochemical properties of membrane lipids and proteins do not adapt fast enough.

## Data Availability Statement

All datasets analysed in this study can be found in the article/Supplementary Material.

## Author Contributions

PL and LL designed the study and collected samples. PL performed respirometry analyses while MW, PT, and RK performed lipid analyses in the HiLIPID/HiLIFE facility. PL wrote the first draft which all authors co-edited and approved.

## Conflict of Interest

The authors declare that the research was conducted in the absence of any commercial or financial relationships that could be construed as a potential conflict of interest.

## Abbreviations

AB: Alder leaf beetle
Ster: cholesterol
CPB: Colorado potato beetle
DAG: diacylglycerol
DGC: discontinuous gas exchange
DBI: double bond index
DPB: dynamic phase behavior
ESI-MS: electrospray ionization mass spectrometry
HPTLC: high-performance thin-layer chromatography
HPA: homeophasic adaptation
HVA: homeoviscous adaptation
MB: Mint or Knotgrass leaf beetle
PC: phosphatidylcholines
PE: phosphatidylethanolamines
PI: phosphatidylinositols
PS: phosphatidylserines
PUFA: polyunsaturated fatty acids
SIMCA: soft independent modeling of class analogies
SM: sphingomyelins
PCA: principal component analyses
SE: sterol esters
TAG: triacylglycerols.

## References

[B1] Alyokhin A. (2009). Colorado potato beetle management on potatoes: current challenges and future prospects. *Fruit Veg. Cereal Sci. Biotechnol.* 3 10–19.

[B2] Arrese E. L. Soulages J. L. (2010). Insect fat body: energy, metabolism, and regulation. *Annu. Rev. Entomol.* 55 207–225. 10.1146/annurev-ento-112408-085356 19725772 PMC3075550

[B3] Athenstaedt K. Daum G. (2006). The life cycle of neutral lipids: synthesis, storage and degradation. *Cell. Mol. Life Sci.* 63 1355–1369. 10.1007/s00018-006-6016-8 16649142 PMC11136409

[B4] Bai Y. McCoy J. G. Levin E. J. Sobrado P. Rajashankar K. R. Fox B. G. (2015). X-ray structure of a mammalian stearoyl-CoA desaturase. *Nature* 524 252–256. 10.1038/nature14549 26098370 PMC4689147

[B5] Bale J. S. Hayward S. A. L. (2010). Insect overwintering in a changing climate. *J. Exp. Biol.* 213 980–994. 10.1242/jeb.037911 20190123

[B6] Ballweg S. Ernst R. (2017). Control of membrane fluidity: the OLE pathway in focus. *Biol. Chem.* 398 215–228. 10.1515/hsz-2016-0277 27787227

[B7] Beekman M. Stratum P. (1999). Respiration in bumblebee queens: effect of life phase on the discontinuous ventilation cycle. *Entomol. Exp. Appl.* 92 295–298. 10.1046/j.1570-7458.1999.00550.x

[B8] Bhat G. B. Block E. R. (1992). Effect of hypoxia on phospholipid metabolism in porcine pulmonary artery endothelial cells. *Am. J. Physiol. Lung. Cell. Mol. Physiol.* 262 L606–L613. 10.1152/ajplung.1992.262.5.L606 1590410

[B9] Boiteau G. Coleman W. (1996). Cold tolerance in the Colorado potato beetle (Leptinotarsa decemlineata) (Say) (Coleoptera: Chrysomelidea). *Can. Entomol.* 128 1087–1099. 10.4039/Ent1281087-6

[B10] Brügger B. Erben G. Sandhoff R. Wieland F. T. Lehmann W. D. (1997). Quantitative analysis of biological membrane lipids at the low picomole level by nano-electrospray ionization tandem mass spectrometry. *Proc. Natl. Acad. Sci. U.S.A.* 94 2339–2344. 10.1073/pnas.94.6.2339 9122196 PMC20089

[B11] Chown S. L. Gibbs A. G. Hetz S. K. Klok C. J. Lighton J. R. B. Marais E. (2006). Discontinuous gas exchange in insects: a clarification of hypotheses and approaches. *Physiol. Biochem. Zool.* 79 333–343. 10.1086/499992 16555192

[B12] Ciancio J. (2018). Overwintering Biology of the Brown Marmorated Stink Bug, Halyomorpha halys (Hemiptera: Pentatomidae). Available online at: https://ir.lib.uwo.ca/etd/5813 (accessed August 20, 2020).

[B13] Corbalán-García S. Gómez-Fernández J. C. (2014). Classical protein kinases C are regulated by concerted interaction with lipids: the importance of phosphatidylinositol-4,5-bisphosphate. *Biophys. Rev.* 6 3–14. 10.1007/s12551-013-0125-z 28509956 PMC5427809

[B14] Danks H. V. (1987). *Insect Dormancy: An Ecological Perspective.* Ottawa: Biological Survey of Canada.

[B15] De Kort C. A. D. Koopmanschap A. B. (1994). Nucleotide and deduced amino acid sequence of a cDNA clone encoding diapause protein 1, an arylphorin-type storage hexamer of the Colorado potato beetle. *J. Insect Physiol.* 40 527–535. 10.1016/0022-1910(94)90126-0

[B16] Denlinger D. L. Lee R. E. J. (2010). *Low Temperature Biology of Insects.* Cambridge: Cambridge University Press.

[B17] Dingle H. (ed.) (1978). *Evolution of Insect Migration and Diapause.* Berlin: Springer.

[B18] EPPO (2006). *Data sheets on quarantine pests - Leptinotarsa decemlineata.* Paris: EPPO.

[B19] Folch J. M. Lees M. Sloane-Stanley G. H. (1957). A simple method for the isolation and purification of total lipides from animal tissue. *J. Biol. Sci.* 497–509. 13428781

[B20] Fründ J. Zieger S. L. Tscharntke T. (2013). Response diversity of wild bees to overwintering temperatures. *Oecologia* 173 1639–1648. 10.1007/s00442-013-2729-1 23864252

[B21] Große W. Schröder P. (1984). Oxygen supply of roots by gas transport in alder-trees. *Z. Naturforsch. C* 39 1186–1188. 10.1515/znc-1984-11-1234

[B22] Guppy M. Wither P. (1999). Metabolic depression in animals: physiological perspectives and biochemical generealizations. *Biol. Rev.* 74 1–40. 10.1111/j.1469-185x.1999.tb00180.x 10396183

[B23] Hahn D. A. Denlinger D. L. (2007). Meeting the energetic demands of insect diapause: Nutrient storage and utilization. *J. Insect Physiol.* 53 760–773. 10.1016/j.jinsphys.2007.03.018 17532002

[B24] Hahn D. A. Denlinger D. L. (2011). Energetics of insect diapause. *Annu. Rev. Entomol.* 56 103–121. 10.1146/annurev-ento-112408-085436 20690828

[B25] Haimi P. Uphoff A. Hermansson M. Somerharju P. (2006). Software tools for analysis of mass spectrometric lipidome data. *Anal. Chem.* 78 8324–8331. 10.1021/ac061390w 17165823

[B26] Hazel J. R. (1995). Thermal adaptation in biological membranes: Is homeoviscous adaptation the explanation? *Annu. Rev. Physiol.* 57 19–42. 10.1146/annurev.ph.57.030195.000315 7778864

[B27] Hiiesaar K. Kaart T. Williams I. H. Luik A. Metspalu L. Ploomi A. (2018). Dynamics of Supercooling Ability and Cold Tolerance of the Alder Beetle (Coleoptera: Chrysomelidae). *Environ. Entomol.* 47 1024–1029. 10.1093/ee/nvy075 29850836

[B28] Hodek I. (2012). Adult Diapause in Coleoptera. *Psyche* 2012 1–10. 10.1155/2012/249081

[B29] Irwin J. T. Lee R. E. J. (2003). Cold winter microenvironments conserve energy and improve overwintering survival and potential fecundity of the goldenrod gall fly, Eurosta solidaginis. *Oikos* 100 71–78. 10.1034/j.1600-0706.2003.11738.x 11841302

[B30] Izzo V. M. Hawthorne D. J. Chen Y. H. (2014). Geographic variation in winter hardiness of a common agricultural pest, Leptinotarsa decemlineata, the Colorado potato beetle. *Evol. Ecol.* 28 505–520. 10.1007/s10682-013-9681-8

[B31] Jõgar K. Kuusik A. Metspalu L. Hiiesaar K. Luik A. Mänd M. (2004). The relations between the patterns of gas exchange and water loss in diapausing pupae of large white butterfly Pieris brassicae (Lepidoptera: Pieridae). *Eur. J. Entomol.* 101 467–472. 10.14411/eje.2004.066

[B32] Johnson C. G. (1967). International dispersal of insects and insect-borne viruses. *Neth. J. Plant Pathol.* 73 21–43. 10.1007/BF01974421

[B33] Kates M. (1986). *Techniques of Lipidology.* Amsterdam: Elsevier.

[B34] Kay J. G. Fairn G. D. (2019). Distribution, dynamics and functional roles of phosphatidylserine within the cell. *Cell Commun. Signal.* 17:126. 10.1186/s12964-019-0438-z 31615534 PMC6792266

[B35] Kölsch G. Jakobi K. Wegener G. Braune H. J. (2002). Energy metabolism and metabolic rate of the alder leaf beetle *Agelastica alni* (L.) (Coleoptera, Chrysomelidae) under aerobic and anaerobic conditions: a microcalorimetric study. *J. Insect Physiol.* 48 143–151. 10.1016/S0022-1910(01)00158-5 12770113

[B36] Kort C. A. D. (1990). Thirty-five years of diapause research with the Colorado potato beetle. *Entomol. Exp. Appl.* 56 1–13. 10.1111/j.1570-7458.1990.tb01376.x

[B37] Koštál V. (2006). Eco-physiological phases of insect diapause. *J. Insect Physiol* 113–127. 10.1016/j.jinsphys.2005.09.008 16332347

[B38] Koštál V. (2010). “Cell structural modifications in insects at low temperatures,” in *Low Temperature Biology of Insects*, eds Denlinger D. L. Lee R. E. J. (Cambridge: Cambridge University Press), 116–132. 10.1017/cbo9780511675997.006

[B39] Koštál V. Štìtina T. Poupardin R. Korbelová J. Bruce A. W. (2017). Conceptual framework of the eco-physiological phases of insect diapause development justified by transcriptomic profiling. *Proc. Natl. Acad. Sci. U.S.A.* 114 8532–8537. 10.1073/pnas.1707281114 28720705 PMC5559046

[B40] Koštál V. Urban T. Øimnáèová L. Berková P. Šimek P. (2013). Seasonal changes in minor membrane phospholipid classes, sterols and tocopherols in overwintering insect, *Pyrrhocoris apterus*. *J. Insect Physiol.* 59 934–941. 10.1016/j.jinsphys.2013.06.008 23845405

[B41] Lal R. (ed.) (2017). *Encyclopedia of Soil Science.* Boca Raton, FL: CRC Press.

[B42] Leather S. R. (1993). *The Ecology of Insect Overwintering.* Cambridge: Cambridge University Press.

[B43] Lefevere K. S. De Kort C. A. D. (1989). Adult diapause in the Colorado potato beetle, Leptinotarsa decemlineata: effects of external factors on maintenance, termination and post-diapause development. *Physiol. Entomol.* 14 299–308. 10.1111/j.1365-3032.1989.tb01097.x

[B44] Lefevere K. S. Koopmanschap A. B. De Kort C. A. D. (1989). Changes in the concentrations of metabolites in haemolymph during and after diapause in female Colorado potato beetle, Leptinotarsa decemlineata. *J. Insect Physiol.* 35 121–128. 10.1016/0022-1910(89)90045-0

[B45] Lehmann P. Lyytinen A. Sinisalo T. Lindström L. (2012). Population dependent effects of photoperiod on diapause related physiological traits in an invasive beetle (Leptinotarsa decemlineata). *J. Insect Physiol.* 58 1146–1158. 10.1016/j.jinsphys.2012.06.003 22705255

[B46] Lehmann P. (2013). *Eco-physiological Aspects of Adaptation to Seasonal Environments: The Latitudinal Range Expansion of the Colorado potato beetle Across Europe.* Available online at: http://urn.fi/URN:ISBN:978-951-39-5364-5 (accessed August 15, 2020).

[B47] Lehmann P. Lyytinen A. Piiroinen S. Lindström L. (2014a). Northward range expansion requires synchronization of both overwintering behavior and physiology with photoperiod in the invasive Colorado potato beetle (Leptinotarsa decemlineata). *Oecologia* 176 57–68. 10.1007/s00442-014-3009-4 25012598

[B48] Lehmann P. Piiroinen S. Kankare M. Lyytinen A. Paljakka M. Lindström L. (2014b). Photoperiodic effects on diapause-associated gene expression trajectories in European *Leptinotarsa decemlineata* populations. *Insect Mol. Biol.* 23 566–578. 10.1111/imb.12104 24924142

[B49] Lehmann P. Kaunisto S. Koštál V. Margus A. Zahradníèková H. Lindström L. (2015a). Comparative ecophysiology of cold-tolerance-related traits: assessing range expansion potential for an invasive insect at high latitude. *Physiol. Biochem. Zool.* 3 254–265. 10.1086/680384 25860825

[B50] Lehmann P. Piiroinen S. Lyytinen A. Lindström L. (2015b). Responses in metabolic rate to changes in temperature in diapausing Colorado potato beetle *Leptinotarsa decemlineata* from three European populations. *Physiol. Entomol.* 40 123–130. 10.1111/phen.12095

[B51] Lehmann P. Lyytinen A. Piiroinen S. Lindström L. (2015c). Latitudinal differences in diapause related photoperiodic responses of European Colorado potato beetles (*Leptinotarsa decemlineata*). *Evol. Ecol.* 29 269–282. 10.1007/s10682-015-9755-x.

[B52] Lehmann P. Pruisscher P. Posledovich D. Carlsson M. Käkelä R. Tang P. (2016). Energy and lipid metabolism during direct and diapause development in a pierid butterfly. *J. Exp. Biol.* 219 3049–3060. 27445351 10.1242/jeb.142687

[B53] Lehmann P. Pruisscher P. Koštál V. Moos M. Šimek P. Nylin S. (2018). Metabolome dynamics of diapause in the butterfly *Pieris napi*: distinguishing maintenance, termination and post-diapause phases. *J. Exp. Biol.* 221:jeb169508. 10.1242/jeb.169508 29180603

[B54] Lehmann P. Ammunét T. Barton M. Battisti A. Eigenbrode S. D. Jepsen J. U. (2020). Complex responses of global insect pests to climate warming. *Front. Ecol. Environ.* 18 141–150. 10.1002/fee.2160

[B55] Lewis R. N. A. H. Mannock D. A. McElhaney R. N. Turner D. C. Gruner S. M. (1989). Effect of fatty acyl chain length and structure on the lamellar gel to liquid-crystalline and lamellar to reversed hexagonal phase transitions of aqueous phosphatidylethanolamine dispersions. *Biochemistry* 28 541–548. 10.1021/bi00428a020 2713331

[B56] Lighton J. B. R. (2008). *Measuring Metabolic Rates: A Manual for Scientists.* New York, NY: Oxford University Press.

[B57] Lighton J. R. B. (1998). Notes from underground: Towards ultimate hypotheses of cyclic, discontinuous gas-exchange in tracheate arthropods. *Am. Zool.* 38 483–491. 10.1093/icb/38.3.483 31919651

[B58] Lindestad O. Schmalensee L. Lehmann P. Gotthard K. (2020). Variation in butterfly diapause duration in relation to voltinism suggests adaptation to autumn warmth, not winter cold. *Funct. Ecol.* 34 1029–1040. 10.1111/1365-2435.13525

[B59] Lindström L. Lehmann P. (2015). “Climate change effects on agricultural insect pests in Europe,” in *Climate Change and Insect Pests*, eds Björkman C. Niemelä P. (Wallingford: CABI), 136–153. 10.1079/9781780643786.0136

[B60] Lyytinen A. Mappes J. Lindström L. (2012). Variation in Hsp70 levels after cold shock: signs of evolutionary responses to thermal selection among *Leptinotarsa decemlineata* populations. *PLoS ONE* 7:e31446. 10.1371/journal.pone.0031446 22319631 PMC3271087

[B61] Marèelja S. (1976). Lipid-mediated protein interaction in membranes. *Biochim. Biophys. Acta* 455 1–7. 990322 10.1016/0005-2736(76)90149-8

[B62] Matthews P. G. D. White C. R. (2011). Discontinuous gas exchange in insects: Is it all in their heads? *Am. Nat.* 177 130–134. 10.1086/657619 21087153

[B63] May M. L. (1989). Oxygen consumption by adult Colorado potato beetles, Leptinotarsa decemlineata (Say) (Coleoptera: Chrysomelidae). *J. Insect Physiol.* 35 797–804. 10.1016/0022-1910(89)90138-8

[B64] Murphy R. C. James P. F. McAnoy A. M. Krank J. Duchoslav E. Barkley R. M. (2007). Detection of the abundance of diacylglycerol and triacylglycerol molecular species in cells using neutral loss mass spectrometry. *Anal. Biochem.* 366 59–70. 10.1016/j.ab.2007.03.012 17442253 PMC2034497

[B65] Nickels J. D. Smith M. D. Alsop R. J. Himbert S. Yahya A. Cordner D. (2019). Lipid rafts: buffers of cell membrane physical properties. *J. Phys. Chem. B* 123 2050–2056. 10.1021/acs.jpcb.8b12126 30605612

[B66] Noronha C. Cloutier C. (1998). Effects of soil conditions and body size on digging by prediapause Colorado potato beetles. *Can. J. Zool.* 76 1705–1713.

[B67] Piiroinen S. Ketola T. Lyytinen A. Lindström L. (2011). Energy use, diapause behaviour and northern range expansion potential in the invasive Colorado potato beetle: Energy use and diapause behaviour. *Funct. Ecol.* 25 527–536. 10.1111/j.1365-2435.2010.01804.x

[B68] Ploomi A. Kuusik A. Jõgar K. Metspalu L. Hiiesaar K. Karise R. (2018). Variability in metabolic rate and gas exchange patterns of the Colorado potato beetle of winter and prolonged diapauses: gas exchange patterns of Colorado potato beetle. *Physiol. Entomol.* 43 251–258. 10.1111/phen.12254

[B69] Raclot T. (2003). Selective mobilization of fatty acids from adipose tissue triacylglycerols. *Prog. Lipid Res.* 42 257–288. 10.1016/S0163-7827(02)00066-8 12689620

[B70] Raclot T. Holm C. Langin D. (2001). Fatty acid speci?city of hormone-sensitive lipase: implication in the selective hydrolysis of triacylglycerols. *J. Lipid Res.* 2049–2057. 11734578

[B71] Ragland G. J. Almskaar K. Vertacnik K. L. Gough H. M. Feder J. L. Hahn D. A. (2015). Differences in performance and transcriptome-wide gene expression associated with Rhagoletis (Diptera: Tephritidae) larvae feeding in alternate host fruit environments. *Mol. Ecol.* 24 2759–2776. 25851077 10.1111/mec.13191

[B72] Rao K. Lakshminarayana G. (1988). Lipid class and fatty acid composition of edible tissues of *Peucedanim graveolens*, *Mentha arvensis*, and *Colocasia esculenta* plants. *J. Agric. Food Chem.* 36 475–578. 10.1021/jf00081a017

[B73] Rozsypal J. Koštál V. Berková P. Zahradníèková H. Šimek P. (2014). Seasonal changes in the composition of storage and membrane lipids in overwintering larvae of the codling moth, *Cydia pomonella* *J. Therm. Biol.* 45 124–133. 10.1016/j.jtherbio.2014.08.011 25436961

[B74] Rozsypal J. Koštál V. Zahradníèková H. Šimek P. (2013). Overwintering strategy and mechanisms of cold tolerance in the codling moth (*Cydia pomonella*). *PLoS One* 8:e61745. 10.1371/journal.pone.0061745 23613923 PMC3629207

[B75] Schmidt-Nielsen K. (1990). *Animal Physiology: Adaptation and Environment.* Cambridge: Cambridge University Press.

[B76] Schneiderman H. Williams C. (1953). The physiology of insect diapause. VII. The respiratory metabolism of the Cecropia silkworm during diapause and development. *Biol. Bull.* 105 320–334.

[B77] Silfverberg H. (2011). Enumeratio renovata Coleopterorum Fennoscandiae, Daniae et Baltiae. *Sahlbergia* 16 1–144.

[B78] Sinclair B. J. (2015). Linking energetics and overwintering in temperate insects. *J. Therm. Biol.* 54 5–11. 10.1016/j.jtherbio.2014.07.007 26615721

[B79] Sinensky M. (1974). Homeoviscous adaptation: a homeostatic process that regulates the viscosity of membrane lipids in *Escherichia coli*. *Proc. Natl. Acad. Sci. U.S.A.* 71 522–525. 10.1073/pnas.71.2.522 4360948 PMC388039

[B80] Šlachta M. Berková P. Vambera J. Koštál V. (2002). Physiology of cold-acclimation in non-diapausing adults of *Pyrrhocoris apterus* (Heteroptera). *Eur. J. Entomol.* 99 181–187. 10.14411/eje.2002.026

[B81] Sokal R. R. Rohlf F. J. (2003). *Biometry: The Principles and Practice of Statistics in Biological Research.* New York, NY: W.H. Freeman and Company.

[B82] Stålhandske S. Lehmann P. Pruisscher P. Leimar O. (2015). Effect of winter cold duration on spring phenology of the orange tip butterfly. Anthocharis cardamines *Ecol. Evol.* 5 5509–5520. 27069602 10.1002/ece3.1773 PMC4813107

[B83] Tauber M. J. Tauber C. A. Masaki S. (1986). *Seasonal Adaptations of Insects.* Oxford: Oxford University Press.

[B84] Toprak U. Hegedus D. Doðan C. Güney G. (2020). A journey into the world of insect lipid metabolism. *Arch. Insect Biochem. Physiol.* 104 1–67. 10.1002/arch.21682 32335968

[B85] Tougeron K. (2019). Diapause research in insects: historical review and recent work perspectives. *Entomol. Exp. Appl.* 167 27–36. 10.1111/eea.12753

[B86] Toxopeus J. Sinclair B. J. (2018). Mechanisms underlying insect freeze tolerance. *Biol. Rev.* 93 1891–1914. 10.1111/brv.12425 29749114

[B87] Urbanski J. Mogi M. O’Donnell D. DeCotiis M. Toma T. Armbruster P. (2012). Rapid adaptive evolution of photoperiodic response during invasion and range expansion across a climatic gradient. *Am. Nat.* 179 490–500. 22437178 10.1086/664709

[B88] Ushatinskaya R. S. (1977). Seasonal migration of the imago of the Colorado potato beetle (Leptinotarsa decemlineata Say) in different types of soil and the physiological variations of specimens in hibernating populations. *Ecol. Bull.* 25 526–529.

[B89] Vrablik T. L. Watts J. L. (2013). Polyunsaturated fatty acid derived signaling in reproduction and development: insights from *Caenorhabditis elegans* and *Drosophila melanogaster*. *Mol. Reprod. Dev.* 80 244–259. 10.1002/mrd.22167 23440886 PMC4350910

[B90] Williams C. Hellmann J. Sinclair B. (2012). Lepidopteran species differ in susceptibility to winter warming. *Clim. Res.* 53 119–130. 10.3354/cr01100

[B91] Williams C. M. Chick W. D. Sinclair B. J. (2015). A cross-seasonal perspective on local adaptation: metabolic plasticity mediates responses to winter in a thermal-generalist moth. *Funct. Ecol.* 29 549–561. 10.1111/1365-2435.12360

[B92] Williams C. M. Henry H. A. L. Sinclair B. J. (2014). Cold truths: How winter drives responses of terrestrial organisms to climate change. *Biol. Rev.* 90 214–235. 24720862 10.1111/brv.12105

[B93] Williams C. M. McCue M. D. Sunny N. E. Szejner-Sigal A. Morgan T. J. Allison D. B. (2016). Cold adaptation increases rates of nutrient flow and metabolic plasticity during cold exposure in Drosophila melanogaster. *Proc. Biol. Sci.* 283:20161317. 10.1098/rspb.2016.1317 PMC5031658 27605506

[B94] Wodtke E. (1981). Temperature adaptation of biological membranes. *Biochim. Biophys. Acta* 640 710–720. 6260175 10.1016/0005-2736(81)90101-2

[B95] Wold S. Sjöström M. (1977). “SIMCA: a method for analyzing chemical data in terms of similarity and analogy,” in*Chemometrics: Theory and Application*, ed. Kowalski B. R. (Washington, DC: American Chemical Society).

[B96] Xu X. Akbar S. Shrestha P. Venugoban L. Devilla R. Hussain D. (2020). A synergistic genetic engineering strategy induced triacylglycerol accumulation in potato (*Solanum tuberosum*) leaf. *Front. Plant Sci.* 11:215. 10.3389/fpls.2020.00215. 32210994 PMC7069356

[B97] Yocum G. D. Buckner J. S. Fatland C. L. (2011a). A comparison of internal and external lipids of nondiapausing and diapause initiation phase adult Colorado potato beetles, Leptinotarsa decemlineata. *Comp. Biochem. Physiol. B Biochem. Mol. Biol.* 159 163–170. 10.1016/j.cbpb.2011.03.007 21496494

[B98] Yocum G. D. Rinehart J. P. Larson M. L. (2011b). Monitoring diapause development in the Colorado potato beetle, Leptinotarsa decemlineata, under field conditions using molecular biomarkers. *J. Insect Physiol.* 57 645–652. 21075113 10.1016/j.jinsphys.2010.11.008

[B99] Zehmer J. K. (2005). Thermally induced changes in lipid composition of raft and non-raft regions of hepatocyte plasma membranes of rainbow trout. *J. Exp. Biol.* 208 4283–4290. 10.1242/jeb.01899 16272251

