# Supplementary material for: The Diapause Lipidomes of Three Closely Related Beetle Species Reveal Mechanisms for Tolerating Energetic and Cold Stress in High-Latitude Seasonal Environments
Source: Front Physiol. 2020 Sep 25;11:576617. doi: 10.3389/fphys.2020.576617 (PMC7546402; doi:10.3389/fphys.2020.576617)
Supplement: Supplementary file 2 [file Table_1.docx]

**The diapause lipidomes of three closely related beetle species reveal mechanisms for tolerating energetic and cold stress in high-latitude seasonal environments**

**Philipp Lehmann^1,2*^, Melissa Westberg^3^, Patrik Tang^3,4^, Leena Lindström^2^, Reijo Käkelä^3,5^**

^1^Department of Zoology, 10691, Stockholm University, Sweden

^2^Department of Biological and Environmental Science, 40014, University of Jyväskylä, Finland

^3^Molecular and Integrative Biosciences Research Programme, Faculty of Biological and Environmental Sciences, University of Helsinki, Finland

^4^Department of Biological Sciences, University of Bergen, Norway

^5^Helsinki University Lipidomics Unit (HiLIPID), Helsinki Institute for Life Science (HiLIFE) and Biocenter Finland, Helsinki, Finland

***Corresponding Author:** [philipp.lehmann@zoologi.su.se](mailto:philipp.lehmann@zoologi.su.se)


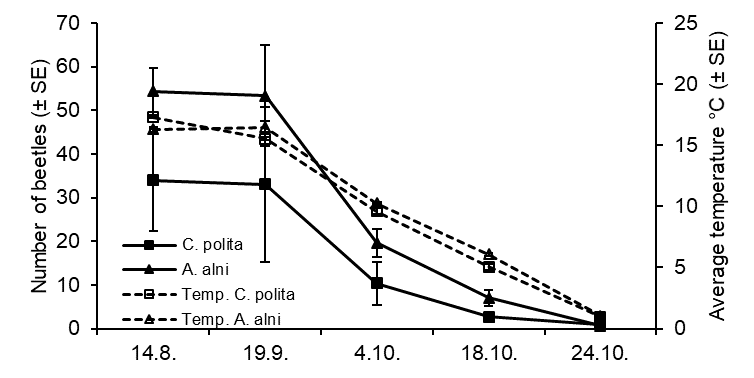
**Supplementary Figure 1.** Field data representing Mint or Knotgrass leaf beetle (MB, *Chrysolina polita*) and Alder leaf beetle (AB, *Agelastica alni*) occurrences in the collection locations. The primary y-axis shows the number of beetles found during a 10-minute search (of 5 trees) in 3 separate plots per date (solid lines). Temperature (dashed lines) was logged at the sampling locations, and represents the average over 1 hour during mid-day.

**Supplementary Figure 2.** Lipid class profiles of adult Colorado potato beetle (CPB, *Leptinotarsa decemlineata*), Mint or Knotgrass leaf beetle (MB, *Chrysolina polita*) and Alder leaf beetle (AB, *Agelastica alni*), which were sampled at three time-points during winter. The first prediapause time-point was when the temperature had been lowered from 23°C to 15°C and adults had stopped feeding, but they had not yet burrowed to the soil (0-month time-point, thus marked 0). The second time-point was two months and the third time-point four months after they had burrowed to the soil (2- and 4-month time-points, thus marked 2 and 4). Each sample group consisted of 14 beetles, 7 females and 7 males. All numbers are expressed as mean ± standard deviation.

**Supplementary Figure 3.** Triacylglycerol species profiles divided into panel A, including C48-52 species, and panel B, including C53-55 species, of adult Colorado potato beetle (CPB, *Leptinotarsa decemlineata*), Mint or Knotgrass leaf beetle (MB, *Chrysolina polita*) and Alder leaf beetle (AB, *Agelastica alni*), which were sampled at three time-points during winter. The first prediapause time-point was when the temperature had been lowered from 23°C to 15°C and adults had stopped feeding, but they had not yet burrowed to the soil (0-month time-point, thus marked 0). The second time-point was two months and the third time-point four months after they had burrowed to the soil (2- and 4- month time-points, thus marked 2 and 4). Each sample group consisted of 12 beetles, 6 females and 6 males. All numbers are expressed as mean ± standard deviation.

**Supplementary Figure 4.** Phosphatidylcholine species profiles of adult Colorado potato beetle (CPB, *Leptinotarsa decemlineata*), Mint or Knotgrass leaf beetle (MB, *Chrysolina polita*) and Alder leaf beetle (AB, *Agelastica alni*), which were sampled at three time-points during winter. The first prediapause time-point was when the temperature had been lowered from 23°C to 15°C and adults had stopped feeding, but they had not yet burrowed to the soil (0-month time-point, thus marked 0). The second time-point was two months and the third time-point four months after they had burrowed to the soil (2- and 4-month time-points, thus marked 2 and 4). Each sample group consisted of 12 beetles, 6 females and 6 males. All numbers are expressed as mean ± standard deviation.

**Supplementary Figure 5.** Sphingomyelin species profiles of adult Colorado potato beetle (CPB, *Leptinotarsa decemlineata*), Mint or Knotgrass leaf beetle (MB, *Chrysolina polita*) and Alder leaf beetle (AB, *Agelastica alni*), which were sampled at three time-points during winter. The first prediapause time-point was when the temperature had been lowered from 23°C to 15°C and adults had stopped feeding, but they had not yet burrowed to the soil (0-month time-point, thus marked 0). The second time-point was two months and the third time-point four months after they had burrowed to the soil (2- and 4- month time-points, thus marked 2 and 4). Each sample group consisted of 12 beetles, 6 females and 6 males. All numbers are expressed as mean ± standard deviation.

**Supplementary Figure 6.** Phosphatidylethanolamine species profiles of adult Colorado potato beetle (CPB, *Leptinotarsa decemlineata*), Mint or Knotgrass leaf beetle (MB, *Chrysolina polita*) and Alder leaf beetle (AB, *Agelastica alni*), which were sampled at three time-points during winter. The first prediapause time-point was when the temperature had been lowered from 23°C to 15°C and adults had stopped feeding, but they had not yet burrowed to the soil (0-month time-point, thus marked 0). The second time-point was two months and the third time-point four months after they had burrowed to the soil (2- and 4- month time-points, thus marked 2 and 4). Each sample group consisted of 12 beetles, 6 females and 6 males. All numbers are expressed as mean ± standard deviation.

**Supplementary Figure 7.** Phosphatidylserine species profiles of adult Colorado potato beetle (CPB, *Leptinotarsa decemlineata*), Mint or Knotgrass leaf beetle (MB, *Chrysolina polita*) and Alder leaf beetle (AB, *Agelastica alni*), which were sampled at three time-points during winter. The first prediapause time-point was when the temperature had been lowered from 23°C to 15°C and adults had stopped feeding, but they had not yet burrowed to the soil (0-month time-point, thus marked 0). The second time-point was two months and the third time-point four months after they had burrowed to the soil (2- and 4- month time-points, thus marked 2 and 4). Each sample group consisted of 12 beetles, 6 females and 6 males. All numbers are expressed as mean ± standard deviation.

**Supplementary Figure 8.** Phosphatidylinositol species profiles of adult Colorado potato beetle (CPB, *Leptinotarsa decemlineata*), Mint or Knotgrass leaf beetle (MB, *Chrysolina polita*) and Alder leaf beetle (AB, *Agelastica alni*), which were sampled at three time-points during winter. The first prediapause time-point was when the temperature had been lowered from 23°C to 15°C and adults had stopped feeding, but they had not yet burrowed to the soil (0-month time-point, thus marked 0). The second time-point was two months and the third time-point four months after they had burrowed to the soil (2- and 4- month time-points, thus marked 2 and 4). Each sample group consisted of 12 beetles, 6 females and 6 males. All numbers are expressed as mean ± standard deviation.
