# Supplementary material for: The Diapause Lipidomes of Three Closely Related Beetle Species Reveal Mechanisms for Tolerating Energetic and Cold Stress in High-Latitude Seasonal Environments
Source: Front Physiol. 2020 Sep 25;11:576617. doi: 10.3389/fphys.2020.576617 (PMC7546402; doi:10.3389/fphys.2020.576617)
Supplement: Supplementary file 3 [file Table_2.docx]

## The diapause lipidomes of three closely related beetle species reveal mechanisms for tolerating energetic and cold stress in high-latitude seasonal environments

**Philipp Lehmann^1,2*^, Melissa Westberg^3^, Patrik Tang^3,4^, Leena Lindström^2^, Reijo Käkelä^3,5^**

^1^Department of Zoology, 10691, Stockholm University, Sweden

^2^Department of Biological and Environmental Science, 40014, University of Jyväskylä, Finland

^3^Molecular and Integrative Biosciences Research Programme, Faculty of Biological and Environmental Sciences, University of Helsinki, Finland

^4^Department of Biological Sciences, University of Bergen, Norway

^5^Helsinki University Lipidomics Unit (HiLIPID), Helsinki Institute for Life Science (HiLIFE) and Biocenter Finland, Helsinki, Finland

***Corresponding Author:** [philipp.lehmann@zoologi.su.se](mailto:philipp.lehmann@zoologi.su.se)

**Supplementary Table 1.** Sample sizes used in all experiments.

| **Sampling point** | **Species** | **Sex** | **Respiration**  **sample size** | **HPTLC**  **sample size** | **ESI-MS**  **sample size** |
| --- | --- | --- | --- | --- | --- |
| Diapause initiation | CPB | Female | 9 | 7 | 7 |
|  |  | Male | 7 | 7 | 7 |
|  | MB | Female | 7 | 7 | 7 |
|  |  | Male | 8 | 7 | 7 |
|  | AB | Female | 9 | 7 | 7 |
|  |  | Male | 6 | 7 | 7 |
| Diapause maintenance | CPB | Female | 11 | 6 | 6 |
|  |  | Male | 11 | 6 | 6 |
|  | MB | Female | 4 | 6 | 6 |
|  |  | Male | 4 | 6 | 6 |
|  | AB | Female | 4 | 6 | 6 |
|  |  | Male | 5 | 6 | 6 |
| Diapause termination | CPB | Female | 8 | 6 | 6 |
|  |  | Male | 8 | 6 | 6 |
|  | MB | Female | 11 | 6 | 6 |
|  |  | Male | 11 | 6 | 6 |
|  | AB | Female | 10 | 6 | 6 |
|  |  | Male | 10 | 6 | 6 |

**Supplementary Table 2.** Equipment, sample application, development and detection parameters in the winCATS- program used for HPTLC-analyses.

| **Sample application:** | **Equipment:** | **Automatic TLC Sampler 4, CAMAG** |
| --- | --- | --- |
|  | **Plates:** | **HPTLC Silica Gel 60 F_254_**  **(20x10 cm)** |
|  | Number of tracks: | 14 |
|  | Application volume: | 15 or 20 µl |
|  | Rinsing solvent: | C:M (1:2) |
|  | Syringe: | 25 µl |
|  | Band lenght: | 6.0 mm |
|  | Application mode: | Spray band |
|  | First application position X: | 15.0 mm |
|  | Application position Y: | 8.0 mm |
|  | Distance between tracks: | Automatic |
| **Development:** | **Equipment:** | **Horizontal Developing Chamber 2, CAMAG** |
|  | Solvent front position: | 70 mm |
|  | Drying devices: | Hair dryer and oven (180 °C) |
| **Detection:** | **Equipment:** | **TLC Scanner 3, CAMAG** |
|  | Scan mode: | Single wavelength |
|  | Scan display scaling: | Automatic |
|  | Spectrum mode: | All detected peaks |
|  | Link parameters to previous TLC steps | On |
|  | Scan start/end position: | 2.0 mm/80.0 mm |
|  | Slit dimensions for phospholipid plates: | 6.00 mm x 0.45 mm, Micro |
|  | Slit dimensions for storage lipid plates: | 8-12 mm x 0.90 mm, Macro |
|  | Optimize optical system for maximum: | Resolution |
|  | Scanning speed: | 1 mm/s |
|  | Data resolution: | 25 µm/step |
|  | Wavelength: | 254 nm |
|  | Lamp: | D2 |
|  | Measurement mode: | Absorption |
|  | Optical filter: | Second order |
|  | Detector mode: | Automatic |
|  | Y- position for 0 adjust: | 2.0 mm |
|  | Track # for 0- adjust: | 1 |
|  | Track start/end for quick scan: | Automatic |
|  | Analog offset: | 10 % |
|  | Sensitivity: | Automatic |
|  | Filter factor: | None |
|  | Baseline correction: | None |
|  | Peak threshold minimum slope/height/area: | 5/3 AU/20 |
|  | Integration limits: track start/end position: | 2.0 mm/80.0 mm |

**Supplementary Table 3.** Lipid standards used for HPTLC analysis.

|  | Standard* | Concentration (µM) | On plate (pmol) |
| --- | --- | --- | --- |
| Storage (neutral) lipid mix | TAG 54:3 (18:1/18:1/18:1) | 60 | 1200 |
|  | Cholesterol ester 18:2 | 60 | 1200 |
|  | Cholesterol | 60 | 1200 |
| Phospholipid mix | PC 40:2 (20:1/20:1) | 50 | 1000 |
|  | SM 17:0 (d18:1/17:0) | 10 | 200 |
|  | PE 32:2 (16:1/16:1) | 30 | 600 |
|  | PS 36:2 (18:1/18:1) | 15 | 300 |

*TAG = triacylglycerol, PC = phosphatidylcholine, SM = sphingomyelin, PE = phosphatidyletanolamine, PS = phosphatidylserine

**Supplementary Table 4.** Lipid standards used for ESI-MS.

| Mode | Lipid species* | Concentration, µM | m/z |
| --- | --- | --- | --- |
| Pos | TAG 48:0 (16:0/16:0/16:0) | 60 | 824.8 [M+NH_4_]^+^ |
| Pos | TAG 60:3 (20:1/20:1/20:1) | 60 | 986.9 [M+NH_4_]^+^ |
| Pos | PC 28:2 (14:1/14:1) | 50 | 674.5 [M]^+^ |
| Pos | PC 40:2 (20:1/20:1) | 50 | 842.9 [M]^+^ |
| Pos | PC 44:2 (22:1/22:1) | 50 | 898.1 [M]^+^ |
| Pos | SM 17:0 (d18:1/17:0) ^#^ | 30 | 717.6 [M]^+^ |
| Neg | PE 28:0 (14:0/14:0) | 30 | 634.5 [M]^-^ |
| Neg | PE 32:2 (16:1/16:1) | 30 | 686.5 [M]^-^ |
| Neg | PS 28:0 (14:0/14:0) | 15 | 678.5 [M]^-^ |
| Neg | PI 34:2 (17:1/17:1) | 8 | 833.7 [M]^-^ |

*TAG: triacylglycerol, PC: phosphatidylcholine, PE: phosphatidyletanolamine,

PS: phosphatidylserine, PI: phosphatidylinositol. ^#^  To quantify SM species, SM 17:0, overlapping the beetle SM species, was used as external standard in combination with the internal PC standards.

**Supplementary Table 5.** Equipment, sample application, and positive and negative ion mode parameters used for ESI-MS.

| **Equipment:** | **ESI- MS:** | **Esquire- LC, Bruker** |
| --- | --- | --- |
| **Sample application:** | **Direct injection:** | **Harvard Apparatus 11Plus** |
|  | Syringe: | Gastight Hamilton- Bonaduz, 250 µl |
|  | Flow speed: | 10 µl/min |
|  | Program: | esquireControl^TM^ Version 6.16 |
| **Pos ion mode parameters:** | Capillary voltage: | - 4000 V |
|  | End plate offset: | -500 V |
|  | Nebulizer: | 15.0 psi |
|  | Dry gas/temp: | 5.00 l/min / 200 °C |
|  | Skim 1/2: | 53.7 V/6.0 V |
|  | Cap exit offset: | 83.2 V |
|  | Cap exit: | 136.9 V |
|  | Octopole/Oct RF: | 2.79 V/150.0 Vpp |
|  | Octopole Δ/Lens: | 2.40 V/-5.0 V |
|  | Trap drive/Lens: | 64.4/-60.0 V |
|  | Scan: | 500 ‒ 1100 m/z |
|  | Detector multiplier: | -1700 V |
|  | Detector dynode: | -5.0 kV |
|  | Lens block voltages, Cap exit: | 0.0 V |
|  | Lens block voltages, skimmer 1/2: | 100.0 V/300.0 V |
|  | Lens block voltages, Oct RF: | 0.0 Vpp |
|  | Lens block voltages, Lens 1/2: | 0.0 V/0.0 V |
| **Neg ion mode parameters:** | Capillary voltage: | +3800 V |
|  | End plate offset: | -872 V |
|  | Nebulizer: | 15.0 psi |
|  | Dry gas/temp: | 5.00 l/min / 200 °C |
|  | Skim 1/2: | -37,3 V/-6.0 V |
|  | Cap exit offset: | -83.2 V |
|  | Cap exit: | -120.5 V |
|  | Octopole/Oct RF: | -2.79 V/150.0 Vpp |
|  | Octopole Δ/Lens: | -2.40 V/5.0 V |
|  | Trap drive/Lens: | 64.4/60.0 V |
|  | Scan: | 600 ‒ 1000 m/z |
|  | Detector multiplier/dynode: | -1700 V/+5.0 kV |
|  | Lens block voltages, Cap exit: | 0.0 V |
|  | Lens block voltages, Skimmer 1/2: | -100.0 V/-300.0 V |
|  | Lens block voltages, Oct RF: | 0.0 Vpp |
|  | Lens block voltages, Lens 1/2: | 0.0 V/0.0 V |

**Supplementary Table 6.** Composition of structural membrane lipids (mol%, mean ± SE, N = 38) during the initiation, maintenance ( 2 months from initiation) and termination phase of diapause (4 months from initiation) in three high latitude Chrysomelid beetles: Colorado potato beetle (CPB) *Leptinotarsa decemlineata*, the Mint or Knotgrass leaf beetle (MB) *Chrysolina polita* and the Alder leaf beetle (AB) *Agelastica alni*. To test the statistical significance of the differences in proportions of lipid classes between the different phases of diapause, either the parametric analysis of variance (* p < 0.05) or non-parametric Kruskal-Wallis test (^#^ p < 0.05) were used. The choice of the test depended on the result of the Levene test using p < 0.05. Statistically significant differences in *post hoc*-tests (Sidak for the parametric test, and Kruskal-Wallis for the non-parametric test) are marked with superscript letters following the mean (the means with no common letter differed at p<0.05 level). PC= phosphatidylcholine, SM = sphingomyelin, PE = phosphatidylethanolamine, PS+PI = phosphatidylserine and phosphatidylinositol, Ster = sterol.

|  | | Diapause  initiation | | Diapause maintenance | | Diapause termination | |
| --- | --- | --- | --- | --- | --- | --- | --- |
|  |  | Mean | SE | Mean | SE | Mean | SE |
|  |  |  |  |  |  |  |  |
| CPB | PC | 39.4 | 2.2 | 39.3 | 1.9 | 37.2 | 2.6 |
| (N = 38) | SM | 5.4 | 0.7 | 6.6 | 0.9 | 7.8 | 1.0 |
|  | PE | 31.6 | 1.3 | 31.6 | 0.9 | 32.0 | 1.0 |
|  | PS+PI | 14.6 | 1.5 | 12.2 | 0.7 | 11.9 | 0.9 |
|  | Ster | 9.0 | 0.6 | 10.3 | 0.7 | 11.1 | 0.6 |
|  |  |  |  |  |  |  |  |
| MB | PC | 34.1 | 1.0 | 33.8 | 0.6 | 36.1 | 0.5 |
| (N = 38) | SM* | 4.2 **^b^** | 0.4 | 6.2 **^a^** | 0.4 | 6.2 **^a^** | 0.4 |
|  | PE | 33.6 | 0.9 | 32.3 | 0.6 | 33.7 | 0.7 |
|  | PS+PI | 13.2 | 2.1 | 9.6 | 0.7 | 9.0 | 0.6 |
|  | Ster* | 15.0 **^b^** | 0.8 | 18.2 **^a^** | 0.8 | 15.0 **^b^** | 0.7 |
|  |  |  |  |  |  |  |  |
| AB | PC | 31.5 | 1.4 | 31.7 | 1.3 | 31.7 | 1.4 |
| (N = 38) | SM* | 4.7 **^b^** | 0.2 | 5.4 **^ab^** | 0.2 | 5.9 **^a^** | 0.3 |
|  | PE | 41.5 | 1.8 | 43.3 | 2.0 | 43.4 | 2.0 |
|  | PS+PI^#^ | 11.5 **^a^** | 0.8 | 9.1 **^ab^** | 0.8 | 8.5 **^b^** | 0.7 |
|  | Ster | 10.7 | 0.7 | 10.6 | 0.6 | 10.6 | 0.5 |
